# Tumor-intrinsic metabolic reprogramming and how it drives resistance to anti-PD-1/PD-L1 treatment

**DOI:** 10.20517/cdr.2023.60

**Published:** 2023-09-04

**Authors:** Kyra Laubach, Tolga Turan, Rebecca Mathew, Julie Wilsbacher, John Engelhardt, Josue Samayoa

**Affiliations:** ^1^Computational Oncology, AbbVie, South San Francisco, CA 94080, USA.; ^2^Immuno-Oncology, AbbVie, South San Francisco, CA 94080, USA.; ^3^Immuno-Oncology, AbbVie, Lake County, IL 60064, USA.

**Keywords:** Immunotherapy resistance, tumor-immune microenvironment, immune checkpoint blockade, energy metabolism, amino acid metabolism, lipid metabolism

## Abstract

The development of immune checkpoint blockade (ICB) therapies has been instrumental in advancing the field of immunotherapy. Despite the prominence of these treatments, many patients exhibit primary or acquired resistance, rendering them ineffective. For example, anti-programmed cell death protein 1 (anti-PD-1)/anti-programmed cell death ligand 1 (anti-PD-L1) treatments are widely utilized across a range of cancer indications, but the response rate is only 10%-30%. As such, it is necessary for researchers to identify targets and develop drugs that can be used in combination with existing ICB therapies to overcome resistance. The intersection of cancer, metabolism, and the immune system has gained considerable traction in recent years as a way to comprehensively study the mechanisms that drive oncogenesis, immune evasion, and immunotherapy resistance. As a result, new research is continuously emerging in support of targeting metabolic pathways as an adjuvant to ICB to boost patient response and overcome resistance. Due to the plethora of studies in recent years highlighting this notion, this review will integrate the relevant articles that demonstrate how tumor-derived alterations in energy, amino acid, and lipid metabolism dysregulate anti-tumor immune responses and drive resistance to anti-PD-1/PD-L1 therapy.

## INTRODUCTION

The development of immune checkpoint blockade (ICB) therapies revolutionized cancer treatment across a variety of indications. Immune checkpoints are necessary for the controlled initiation and termination of immune responses as well as for the maintenance of self-tolerance, which are critical in preventing autoimmunity^[1]^. However, tumors leverage this checkpoint system to inappropriately dampen the immune response and facilitate immune escape^[1]^. Continuous antigen stimulation drives the upregulation of checkpoint receptors on CD8^+^ T cells^[2]^, while tumor cells exploit a variety of mechanisms to upregulate checkpoint ligands. Therefore, blocking the interaction between immune checkpoint receptors and ligands reinvigorates CD8^+^ T cell function to elicit tumor cell killing. There are several ICB therapies that are currently utilized in the clinic, but the most well-studied are anti-programmed cell death protein 1 (anti-PD-1), which is predominantly found on T cells, and anti-programmed cell death ligand 1 (anti-PD-L1), which is expressed on tumor and myeloid cells^[3]^. While anti-PD-1/PD-L1 treatments are widely used, a substantial number of patients are resistant to this type of therapy^[4]^, prompting researchers to identify resistance mechanisms that drive inadequate outcomes. Response to ICB is largely dependent on the existing profile and infiltration of immune cells within the tumor, specifically CD8^+^ T cells, because they are the main contributors to anti-tumor effects^[4]^. Therefore, modulating the tumor-immune microenvironment (TIME) to enhance CD8^+^ T cell infiltration and function, in combination with current ICB therapies, serves as an attractive approach to increase efficacy and overcome resistance.

The intersection of cancer and metabolism has been at the forefront of oncology research for several decades. Otto Warburg and his identification of the Warburg effect, wherein malignant cells exhibit a metabolic shift from oxidative phosphorylation to glycolysis^[5]^, ignited massive research efforts towards uncovering the metabolic reprogramming that occurs in tumors. These efforts led to the classification of dysregulated tumor cell metabolism as one of the hallmarks of cancer in 2022^[6]^. Therefore, altered metabolism of lipids, amino acids, carbon, and nucleotides, to name a few, are highly implicated in the development and progression of cancer^[7]^. More recently, this field of onco-metabolism has expanded to include the immune system, given its role in regulating tumorigenesis. Immune cells and their subtypes have different metabolic requirements during activation, differentiation, and expansion^[8]^, wherein alterations in the extrinsic metabolome at any of these stages can lead to immune cell dysfunction. The TIME is an objectively harsh environment for many cell types due to its acidity, hypoxia, nutrient deprivation, and accumulation of inhibitory metabolites^[9]^. To the advantage of the tumor, malignant and immunosuppressive cells, such as T regulatory cells (Tregs), myeloid-derived suppressor cells (MDSCs), and macrophages, are better adapted to this oppressive environment compared to anti-tumor CD8^+^ T cells^[10]^. These conditions, which are largely facilitated by cancer cells, heavily contribute to decreased CD8^+^ T cell infiltration and function.

There is mounting evidence that tumor-intrinsic metabolic reprogramming has a profound effect on the recruitment and function of various immune cell types within the TIME. As such, it is necessary to identify ways to specifically target malignant cell metabolism to enhance the efficacy of ICB. The scope of this review article will aim to cover the current literature that demonstrates how tumor-derived alterations in energy, amino acid, and lipid metabolism within the TIME mediate CD8^+^ T cell dysfunction and how targeting these pathways combats resistance to anti-PD-L1/PD-1 treatment.

## ENERGY METABOLISM

Energy metabolism includes a complex network of biochemical pathways that contribute to sustained cellular function through the production of adenosine triphosphate (ATP). Some of these processes include glycolysis, the tricarboxylic acid (TCA) cycle, and fatty acid b-oxidation. A shift in energy metabolism towards the Warburg effect in malignant cells generates high levels of lactic acid, while consuming and producing ATP/adenosine diphosphate (ADP) and oxidizing and reducing nicotinamide adenine dinucleotide (NAD). While concurrently studying lactate, adenosine, and NAD^+^ in the context of energy metabolism is important, each individual metabolite uniquely influences the function of malignant and immune cells within the TIME. Therefore, this section will focus on how the altered metabolism of lactate, adenosine, and NAD^+^ by tumor cells impacts the anti-tumor immune response by CD8^+^ T cells and contributes to anti-PD-1/PD-L1 resistance.

### Lactate

**Figure 1:**
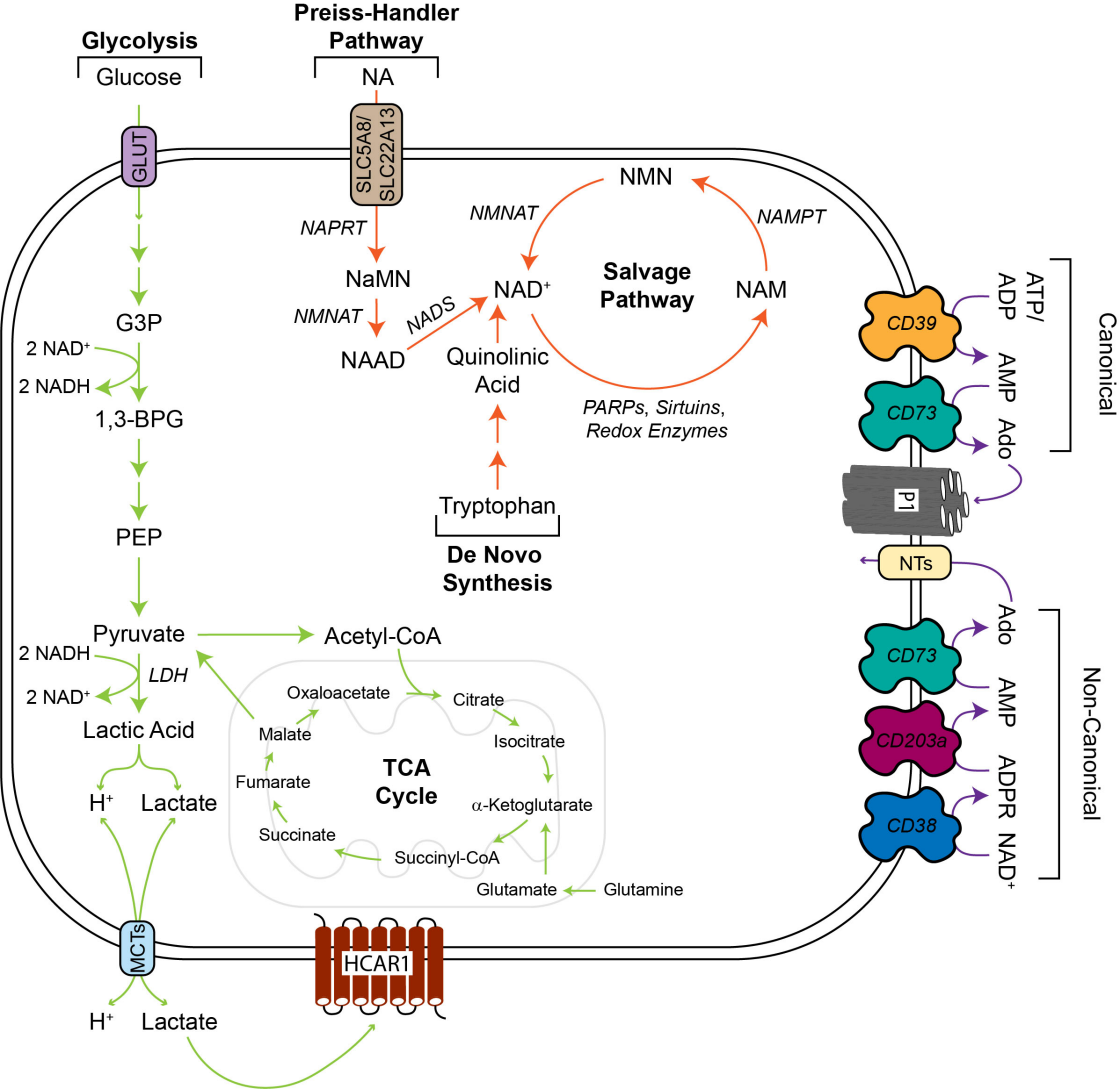
Energy metabolism pathways for lactate, adenosine, and NAD^+^. Pyruvate is generated predominantly through glycolysis, but the TCA cycle also contributes to pyruvate production via conversion from malate. LDH catalyzes the reaction to convert pyruvate to lactic acid, which dissociates into H^+^ and lactate ions that are exported and imported through MCTs. Alternatively, pyruvate can be converted to acetyl-CoA to participate in the TCA cycle to drive energy metabolism. In the TIME, H^+^ contributes to the low pH and lactate facilitates a variety of intracellular signaling pathways by binding to HCAR1. Extracellular adenosine is formed through both the canonical and non-canonical pathways. The canonical pathway utilizes CD39 to convert ATP or ADP to AMP and CD73 to convert AMP to adenosine. The non-canonical pathway metabolizes NAD^+^ to ADPR through CD38, ADPR to AMP through CD203a, and finally, AMP to adenosine via CD73. Extracellular adenosine binds to P1 to initiate intracellular signaling pathways or is imported through NTs. Note: adenosine generated by the canonical and non-canonical pathways participates in both P1 signaling and NT import. NAD^+^ is formed through the Preiss-Handler pathway, de novo synthesis, salvage pathway, and various enzymatic reactions in energy metabolism, such as PEP to pyruvate. The Preiss-Handler pathway imports NA and forms NAD^+^ through a series of enzymatic reactions. Do novo synthesis of NAD^+^ results from the metabolism of tryptophan and the salvage pathway recycles NAM to regenerate intracellular NAD^+^ levels. NAD^+^ serves as a co-factor for many enzymes and participates in redox reactions, such as pyruvate to lactic acid. Ado: Adenosine; ADP: adenosine diphosphate; ADPR: adenosine diphosphate ribose; AMP: adenosine monophosphate; ATP: adenosine triphosphate; GLUT: glucose transporter; G3P: glycerol-3-phosphate; H^+^: hydrogen; HCAR1: hydroxycarboxylic acid receptor 1; LDH: lactate dehydrogenase; MCTs: monocarboxylate transporters; NA: nicotinic acid; NAAD: nicotinic acid adenine dinucleotide; NAD^+^: nicotinamide adenine dinucleotide; NADS: NAD^+^ synthetase; NAM: nicotinamide; NaMN: nicotinic acid mononucleotide; NAMPT: nicotinamide phosphoribosyltransferase; NAPRT: nicotinic acid phosphoribosyltransferase; NMNAT: nicotinamide mononucleotide adenylyltransferase; NMN: nicotinamide mononucleotide; NTs: nucleoside transporters; PARP: poly (ADP-ribose) polymerase; PEP: phosphoenolpyruvate; P1: type 1 purinergic receptors; TCA: tricarboxylic acid; 1,3-BPG: 1,3-Bisphosphoglycerate.

Lactate is predominantly formed through glycolysis, wherein lactate dehydrogenase (LDH) reduces pyruvate to lactic acid, which then dissociates into hydrogen (H^+^) and lactate ions [Figure 1]. To a lesser extent, glutaminolysis also drives pyruvate formation, resulting in lactic acid production^[11]^. Lactate and H^+^ are exported through proton-linked monocarboxylate transporters 1-4 (MCT1-4)^[12]^, wherein export is highly dependent on the existing concentration of extracellular lactate^[13]^. Intracellular lactate levels are also modulated by import through MCT1^[14]^. Extracellular lactate facilitates intracellular signaling by binding to hydroxycarboxylic acid receptor 1 (HCAR1), which regulates a variety of downstream oncogenic pathways, such as cell proliferation, migration, and invasion^[15]^. Accumulation of H^+^ via lactic acid production contributes to the acidity of the TIME, which promotes an immunosuppressive milieu^[16]^. Conversely, lactate ions have both tumor-promoting and -inhibiting effects in CD8^+^ T cells.

T cells require adequate levels of lactic acid for proper development and function^[17,18]^, but excess amounts in the TIME and intracellularly promote dysfunction. Tumor-derived lactic acid accumulation within the TIME inhibits T cell proliferation and cytokine production by altering redox homeostasis^[19]^. Specifically, lactic acid downregulates T cell production of both reactive oxygen species (ROS) and the antioxidant glutathione^[19]^. While excess amounts of ROS promote oxidative stress, low levels are important for T cell activation and signaling^[20]^, suggesting that tumor-derived lactic acid inhibits T cell functions by ablating ROS formation. Additionally, overabundance of lactic acid in the TIME prevents T cell export of lactate and H^+^ ions because of the unfavorable concentration gradient, and subsequent accumulation promotes intracellular acidification and decreases effector function^[21]^. In particular, intracellular acidification in T cells due to tumor-derived lactic acid production prevents the expression of nuclear factor of activated T cells (NFAT)^[22]^, a family of transcription factors that mediate T cell development^[23]^. In CD8^+^ T cells, decreased NFATC1 expression reduces IFNg production, whereas inhibiting lactate dehydrogenase A (LDHA) reduces intracellular acidification and restores CD8^+^ T cell function and tumor infiltration^[22]^. Similarly, the hypoxic nature of the TIME drives upregulation of LDHA in CD8^+^ tumor-infiltrating lymphocytes (TILs), leading to excess intracellular lactic acid, which then inhibits IFNg and granzyme B production^[24]^ and T cell expansion^[18]^. Upon chronic antigen stimulation, CD8^+^ T cells will progress through progenitor exhausted and terminally exhausted states, with the latter resulting in dysfunction and the inability to elicit anti-tumor effects^[25]^. Therefore, there has been a significant focus on promoting the expansion of non-exhausted states and inhibiting the progression into terminal exhaustion to reinvigorate the anti-tumor response. Researchers found that treatment of CD8^+^ T cells with IL-21 promotes expansion but does not drive T cells towards an exhausted state, like IL-2^[18]^. Moreover, IL-2, but not IL-21, induced metabolic reprogramming in T cells to favor glycolysis and shunt pyruvate towards lactic acid formation^[18]^. Treatment with IL-2 and LDH inhibitor invoked a shift from glycolysis towards oxidative phosphorylation, and IL-2 or IL-21 treatment in combination with LDH inhibitor increased stem cell memory T cell formation and reduced tumor growth^[18]^. These data demonstrate that tumor-derived lactic acid can directly or indirectly inhibit T cell function and anti-tumor immune response.

Lactate serves as a carbon source in both tumor and T cells^[26-28]^, but like with any metabolite, overabundance dampens cellular functions. In T cells, increased lactate metabolism depletes NAD^+^ levels by reducing it to NADH, preventing the downstream glycolytic processes that rely on NAD^+^^[29]^. Similarly, reduced glycolytic flux in T cells diminishes serine production, which is critical for T cell proliferation^[29]^. Moreover, tumor-derived lactate promotes depletion of NAD^+^ in naïve T cells, resulting in translation inhibition of FIP200, which forms one subunit of the ULK kinase complex that regulates autophagy^[30]^. FIP200 is selectively lost in naïve T cells from ovarian cancer patients, wherein autophagy is suppressed, leading to mitochondrial dysfunction and, ultimately, apoptosis^[30]^. Genetic ablation of FIP200 in naïve T cells reduced CD8^+^ and CD4^+^ T cell infiltration and IFNg production^[30]^. Recently, tumor-derived lactate was also found to diminish TCA-intermediate recycling in CD8^+^ T cells by inhibiting pyruvate carboxylase, which shunts pyruvate to oxaloacetate^[31]^. Pyruvate carboxylase is exceedingly important to maintain TCA cycle anaplerosis in CD8^+^ T cells because succinate is diverted from the TCA cycle to participate in autocrine signaling^[31]^. In addition to tumor-derived lactate suppressing CD8^+^ T cell function, it also drives the expansion and function of immunosuppressive cells. Tregs inhibit the function of anti-tumor immune cells and require lactate to maintain their suppressor functions in the harsh TIME^[32,33]^. Moreover, lactate produced by cervical cancer cells supports immunosuppressive macrophages by regulating anti-inflammatory cytokine production and HIF1a expression^[34]^. Taken together, these data highlight that tumor-derived lactate not only directly inhibits effector T cell functions, but also indirectly through supporting immunosuppressive cell populations. As such, multiple reports have examined the feasibility of inhibiting tumor-intrinsic lactate metabolism in combination with anti-PD-1/PD-L1 therapy.

Several correlative studies through bioinformatic analyses have demonstrated that targeting lactic acid metabolism might overcome ICB resistance and yield better patient outcomes. High LDH expression has been evaluated as a selection criterion for and predicting response to ICB therapy^[35-39]^. Similarly, other lactate-related genes have been correlated with the expression of immune checkpoint proteins, CD8^+^ T cell infiltration, and resistance to ICB in breast cancer^[40]^. Moreover, decreased glycolytic flux in melanoma patients treated with anti-PD-1 therapy was associated with increased probability of progression-free survival^[41]^.

In addition to bioinformatics studies, numerous reports indicate that inhibiting tumor-intrinsic lactic acid metabolism in combination with anti-PD-1/PD-L1 therapies combats resistance and increases efficacy. MCT4 is regulated at the mRNA level by the demethylase alkB homolog 5 (ALKBH5)^[42]^. Genetic or pharmacologic inhibition of ALKBH5 reduces intratumoral lactate concentration and the number of Tregs and MDSCs, but has no effect on the number of infiltrating cytotoxic T cells^[42]^. Furthermore, utilizing a small molecular inhibitor of ALKBH5 significantly improved the efficacy of anti-PD-1 treatment in murine melanoma tumors^[42]^. Consistent with the findings that lactic acid benefits immunosuppressive cells, researchers found that lactic acid produced by high-glycolytic tumors drove expression of PD-1 on Tregs, but not CD8^+^ T cells, leading to anti-PD-1 resistance^[43]^. However, inhibiting either LDHA in tumors or MCT1 in Tregs combined with anti-PD-1 therapy reversed these effects^[43]^. In addition to inhibiting lactic acid production and/or lactate import, antagonizing intracellular lactate signaling in malignant cells through HCAR1 also promotes anti-tumor effects^[44]^. Abrogating HCAR1-mediated lactate signaling sensitized tumors to anti-PD-1 and metformin treatment, leading to reduced tumor volume and increased CD8^+^ T cell infiltration and IFNg production^[44]^.

While a plethora of evidence supports the notion that lactic acid production by tumors and accumulation in T cells drives oncogenesis, a few reports contradict this idea. In mouse melanoma tumors, blocking the export of lactate and H^+^ ions through MCT1 and MCT4 reduced the acidification of the TIME^[41]^. While blocking MCT1 and 4 in T cells decreased lactate secretion and glucose uptake, it surprisingly did not impair IFNg production^[41]^, which contrasts with other findings that accumulation of intracellular lactic acid promotes acidification and dampens effector functions^[21,22,24]^. The authors found that inhibiting MCT1 and 4 activities in T cells increased glucose flux through the TCA cycle and increased oxygen consumption, thus providing an explanation as to why CD8^+^ T cell effector functions were preserved^[41]^. Moreover, pharmacologically inhibiting MCT1 and 4 in combination with anti-PD-1 treatment resulted in increased efficacy and decreased tumor volume^[41]^. The results from these findings are indeed surprising given the mounting evidence that accumulation of lactic acid within T cells dampens their function. Researchers have also found that lactate, when studied separately from H^+^ in the form of sodium lactate, induces stemness and tumor infiltration, and reduces apoptosis in CD8^+^ T cells^[45]^. Moreover, sodium lactate supplementation in three mouse tumor models showed synergistic effects with anti-PD-1 treatment^[45]^. A plausible explanation for these somewhat contradictory findings is that variations between the TIMEs of different tumor types metabolically reprogram CD8^+^ TILs in distinct ways, wherein some tumors drive increased sensitivity of CD8^+^ TILs to lactic acid. Therefore, it is exceedingly important to delineate the metabolic changes in CD8^+^ TILs from different tumor types to identify the most effective therapy.

Additional research is needed to tease apart the intricate relationship between lactate, lactic acid, tumor cells, CD8^+^ T cells, and immunosuppressive cells. Inhibiting tumor-derived lactic acid production seems to generally have anti-tumor effects, due to the detrimental effects of high acidity on the anti-tumor immune cells within the TIME. While lactate ions serve as a carbon source and promote CD8^+^ T cell stemness, they also benefit immunosuppressive cells and excess amounts can dampen T cell effector functions. Collectively, these data demonstrate that tumor-derived alterations in lactic acid metabolism contribute to ICB resistance and modulating these pathways may augment efficacy, prompting the need for continued research efforts in this field.

### Adenosine

Adenosine is formed through two major pathways [Figure 1]. In the canonical pathway, ectonucleoside triphosphate diphosphohydrolase-1 (CD39) hydrolyzes ATP or ADP to adenosine monophosphate (AMP)^[46]^, which is subsequently converted to adenosine by ecto-5′-nucleotidase (CD73)^[47]^. The non-canonical pathway involves the conversion of NAD^+^ to adenosine diphosphate ribose (ADPR) through cyclic ADP ribose hydrolase (CD38); ADPR is then metabolized to AMP via ectonucleotide pyrophosphatase/phosphodiesterase 1 (CD203a), and finally to adenosine through CD73^[48]^. Extracellular adenosine has several fates; it is converted to inosine via adenosine deaminase, converted back to AMP through adenosine kinase, or binds to type 1 purinergic receptors, which include A1, A2A, A2B, and A3. Both A2A and A2B receptors (A2AR and A2BR) are important for mediating adenosine signaling in immune cells within the TIME^[49]^. High affinity A2AR is more broadly expressed on immune cells, while low affinity A2BR facilitates the expansion of MDSC populations^[50]^.

Within the TIME, adenosine formation is predominantly mediated by malignant and immunosuppressive cells^[51]^ and the impact of this metabolite on immunosuppression and cancer progression was recently comprehensively reviewed^[52]^. Under physiological conditions, extracellular ATP and adenosine levels are low^[53]^. However, during cellular stress, such as hypoxia and nutrient deprivation, intracellular ATP is released and serves as a strong pro-inflammatory mediator by recruiting immune cells^[53,54]^. On the other hand, adenosine is a potent immunosuppressive metabolite^[50]^. As such, it is not surprising that tumor cells highly upregulate CD73 and immunosuppressive cells, such as cancer-associated fibroblasts (CAFs), Tregs, and MDSCs, highly upregulate CD39 to facilitate adenosine accumulation within the TIME^[52,55-59]^. Further, terminally exhausted CD8^+^ T cells exhibit increased CD39 expression, therefore contributing to the elevated adenosine levels within the TIME^[60]^, and adenosine drives the expansion of Treg populations^[61]^.

Tumor-derived adenosine inhibits CD8^+^ T cell functions in a myriad of ways. Adenosine triggers IL-10 secretion from cervical cancer cells, leading to downregulation of MHC-I expression and subsequent immune evasion from CD8^+^ T cells^[62]^. Increased adenosine production also favors tumor growth, as indicated by the negative correlation between CD73 expression and survival in pancreatic adenocarcinoma human cohorts^[63]^. Moreover, loss of CD73 in pancreatic ductal adenocarcinoma cell lines leads to increased activation and IFNg production in CD8^+^ T cells^[63]^, highlighting the inverse relationship between adenosine and CD8^+^ T cell function. Adenosine production within the TIME is also regulated by cancer exosomes, which are endosomal-derived extracellular vesicles^[64,65]^. Specifically, cancer exosomes were found to express CD39 and CD73, leading to inhibition of T cell activation and proliferation in human neuroblastoma samples^[66]^ and bladder, colorectal, prostate, and breast cancer cell lines^[67]^. Accumulation of adenosine within the TIME also severely hinders tumor infiltration by CD8^+^ T cells due to adenosine-mediated dysfunction of KCa3.1 channels^[68,69]^. KCa3.1 is a potassium channel that regulates Ca^2+^ influx, which affects T cell gene expression, activation, and differentiation^[70]^. Inhibition of KCa3.1 by adenosine reduced T cell migration and cytokine production^[69]^, and decreased KCa3.1 channel activity, but not protein expression, resulting in decreased tumor infiltration^[68]^. Building on this, the same group later found that anti-PD-1 therapy increased the activity of ion channels KCa3.1 and Kv1.3, leading to enhanced CD8^+^ T cell infiltration in head and neck squamous cell carcinoma (HNSCC) patient samples^[71]^. While not the focus of this section, it is important to mention that Treg-derived adenosine also drives CD8^+^ T cell dysfunction^[56,57,72,73]^. On the other hand, increased IL-7 signaling in CD8^+^ T cells inhibits FoXO1 activation, which is a transcription factor that controls T cell proliferation, to overcome the suppressive effects of the adenosine-rich TIME and promote tumor infiltration and expansion^[74]^. Leveraging these mechanisms might be a viable therapeutic strategy to be used in conjunction with current ICB therapies to overcome resistance.

Adenosine within the TIME engages with the A2A receptor (A2AR) on CD8^+^ T cells to drive adenosinergic signaling that results in impaired anti-tumor effects^[75]^. Early studies found that A2AR signaling inhibited T cell activation and proliferation^[76]^, and in the context of cancer, many studies have shown that A2AR signaling promotes immune evasion and T cell dysfunction. In mouse melanoma and fibrosarcoma models, pharmacological inhibition or genetic deficiency of A2AR increases CD8^+^ T cell tumor infiltration and IFNg production, and reduces tumor growth^[77,78]^. Moreover, targeted knockdown or antagonizing A2AR increases CD8^+^ T cell infiltration^[79]^ and decreases Treg infiltration and tumor volume in mouse models of HNSCC^[80]^. Similarly, administering A2AR agonists during T cell activation impaired cytotoxic function, although proliferative capacity was maintained, and these effects persisted after A2AR agonists were removed^[81]^. These data demonstrate that even if CD8^+^ T cells infiltrate the adenosine-rich TIME, adenosinergic signaling reduces their effector functions and renders them incapable of eliminating tumor cells. However, one study showed that complete abrogation of the A2AR gene in CD8^+^ T cells inhibited expansion and effector functions^[75]^. In this way, it is important to preserve some degree of A2AR signaling in CD8^+^ T cells to maintain proper cell function, highlighting that complete deletion of immunosuppressive targets might not produce the most efficacious results.

The studies thus far have demonstrated that tumor-intrinsic adenosine metabolism adversely affects CD8^+^ T cell function; therefore, it is not surprising that these metabolic alterations also contribute to anti-PD-1/PD-L1 resistance. To date, there are many drugs in the pre-clinical and clinical stages that target CD39, CD73, and A2AR, either alone or in combination with anti-PD-1/PD-L1 therapies^[82]^. Because it is not feasible to cover all these data, we have chosen to focus on the relevant articles from 2020 until now to demonstrate that modulating adenosine metabolism helps overcome resistance to ICB therapies. Using bioinformatics approaches, researchers showed that adenosine signaling gene signatures are inversely correlated with survival and efficacy of anti-PD-1 treatment across multiple cancer indications^[83]^. The first-in-human study using an A2AR antagonist with anti-PD-L1 treatment improved the probability of progression-free survival and overall survival, and monotherapy or combination with anti-PD-L1 increased CD8^+^ T cell infiltration^[84]^. However, current A2AR antagonists do not perform well in the adenosine-rich TIME, so multiple groups have developed novel A2AR antagonists to increase effectiveness^[85,86]^. Both compounds have shown limited toxicity in Phase I clinical trials^[85,86]^, with iTeos Therapeutics’ compound demonstrating initial signs of clinical benefit^[86]^. Dizal Pharmaceuticals’ compound was also evaluated in murine models of prostate cancer, where treatment with the novel antagonist and anti-PD-1 significantly reduced tumor volume compared to monotherapy^[85]^.

There are several pre-clinical and clinical studies that demonstrate promising results for targeting CD39 or CD73 in combination with anti-PD-1 or PD-L1. Cancer exosomes expressing CD39 and CD73 drive adenosine accumulation and were also found to promote CD39 expression on macrophages^[87]^. Macrophage-derived CD39 cooperates with tumor-derived CD73 to increase adenosine levels in the TIME, which drives anti-PD-1 resistance^[87]^. Targeting CD39 on macrophages in combination with anti-PD-1 therapy abrogated therapeutic resistance and synergistically reduced the volume of murine hepatocellular carcinoma tumors and increased CD8^+^ T cell infiltration and granzyme B production^[87]^. Moreover, a first-in-human Phase I clinical trial was conducted in 2020 to assess the efficacy of an anti-CD39 antibody (IPH5201) in combination with anti-PD-L1 treatment^[88]^, and the first patient for the Phase II study was dosed in June 2023^[89]^. A poster presentation at the European Society for Medical Oncology Immuno-Oncology Summit in 2022 showed pre-clinical data for IPH5201, wherein treatment alone reduced adenosine levels in the TIME of mouse fibrosarcoma tumors^[90]^. The data also demonstrated that combining anti-CD39, the chemotherapeutic agent gemcitabine, and anti-PD-L1 controlled tumor growth and increased survival better than monotherapy or anti-PD-L1 with gemcitabine in murine colorectal carcinoma tumors^[90]^. In a clinical study of 44 patients, researchers found no major toxicities when combining an anti-CD39 monoclonal antibody with anti-PD-1 and the chemotherapy regimen FOLFOX for the treatment of gastric cancer or gastroesophageal junction adenocarcinoma^[91]^. These data are critical first steps in the approval and use of anti-CD39 therapies in combination with anti-PD-1/PD-L1 treatment. The results from a first-in-human Phase I clinical trial with anti-CD73 and anti-PD-L1 recently reported tolerable safety and moderate efficacy^[92]^. Further, targeting CD73 has also recently been shown to be a promising therapeutic strategy, wherein Phase II clinical trials combining anti-CD73 with anti-PD-L1 elicit increased response rate and progression-free survival compared to anti-PD-L1 monotherapy in patients with non-small cell lung cancer^[93]^. One thing to consider when targeting CD39 or CD73 is that anti-CD39 treatments not only inhibit adenosine production, but also promote accumulation of immunostimulatory ATP.

In addition to more conventional treatment methods, several unique approaches for inhibiting adenosine metabolism and PD-1 have recently been discovered. Because of the ubiquitous expression of A2AR on T cells, localizing inhibition of A2AR signaling to tumor-infiltrating CD8^+^ T cells would likely mitigate off-target effects. In this approach, researchers increased tumor oxygenation to relieve the hypoxic conditions that promote tumor-derived adenosine production^[94]^. Using a photo-modulated nanoreactor, hydrogen peroxide is converted to oxygen within the TIME, leading to decreased adenosine production and abrogated A2AR signaling in CD8^+^ T cells^[94]^. Moreover, combination with anti-PD-1 therapy synergistically reduced tumor growth and increased CD8^+^ T cell infiltration in triple-negative murine breast cancer tumors^[94]^. In another tumor-targeting approach, researchers utilized cancer-derived exosomes packaged with both a CD39 antagonist and AMPK agonist to inhibit adenosine and promote ATP production, respectively^[95]^. This method increased CD8^+^ T cell infiltration and production of granzyme B and IFNg, reduced intratumoral adenosine and Treg populations, and synergized with anti-PD-1 treatment in mouse melanoma models^[95]^. The final targeted approach used ROS-producing nanoparticles to deliver a CD39 inhibitor^[96]^. Inducing ROS accumulation in the TIME seems counterintuitive, but like hypoxia, ROS trigger the release of ATP. Therefore, ROS would increase ATP concentration and inhibiting CD39 would prevent adenosine formation, thus remodeling the TIME away from an immunosuppressive state^[96]^. This method alone decreased tumor volume and increased CD8^+^ T cell production of IFNg and, together with anti-PD-1, elicited a more robust anti-tumor effect in murine mammary carcinoma tumors^[96]^.

Collectively, these data strongly demonstrate that tumor-derived adenosine has detrimental effects on CD8^+^ T cell infiltration and effector functions, thereby contributing to anti-PD-1/PD-L1 resistance mechanisms. As such, there is a compelling need for the continued development of adenosine-targeting drugs that can synergize with current anti-PD-1/PD-L1 therapies to prevent resistance and evoke better patient response.

### NAD^+^

NAD^+^ is comprised of adenosine monophosphate linked to nicotinamide mononucleotide. NAD^+^ can be reduced to form NADH or phosphorylated and subsequently reduced to form NADP^+^ or NADPH, respectively. NAD^+^ is synthesized through three pathways: de novo biosynthesis, Preiss-Handler pathway, or the salvage pathway, the latter of which is the predominant way that cells restore NAD^+^ levels^[97]^ [Figure 1]. NAD^+^ is a co-factor that is involved in a variety of redox and non-redox reactions. In energy metabolism, NAD^+^ and its derivatives are indispensable for cellular function because they accept and donate electrons in a variety of metabolic pathways, such as glycolysis, pentose phosphate pathway, TCA cycle, and fatty acid b-oxidation^[98]^. NAD^+^ also acts as a substrate for multiple enzyme families, including sirtuins, PARPs, and ADP-ribosyl cyclases^[97]^. Moreover, the metabolic pathways of adenosine and NAD^+^ are tightly linked through CD38, an ectoenzyme present on the surface of tumor and immune cells, which depletes NAD^+^ levels, which ultimately results in adenosine formation^[99]^.

High NAD^+^ levels are required in malignant cells to meet their increased energetic demands for rapid growth and proliferation. Therefore, malignant cells will upregulate NAD^+^ biosynthesis to replenish intracellular stores, leading to depletion of this metabolite within the TIME. Several enzymes involved in anabolic NAD^+^ pathways, such as nicotinamide phosphoribosyltransferase (NAMPT), have been heavily implicated in cancer progression and severity^[100]^. Moreover, drugs targeting these enzymes have shown promising results in pre-clinical and clinical studies^[101]^. Targeting tumor-intrinsic NAD^+^ metabolism is a promising therapeutic approach because it would restore NAD^+^ levels in the TIME, thus allowing T cells to utilize this metabolite to maintain proper function.

NAD^+^ is highly important for anti-tumor immune functions and NAMPT is an important regulator of NAD^+^ availability. As previously mentioned, NAD^+^ and adenosine metabolism are highly linked due to the ability of NAD^+^ to be converted to adenosine. Inhibiting NAMPT in tumor cells reduces levels of intracellular NAD^+^ and extracellular adenosine, thereby enhancing CD8^+^ T cell functions^[102]^. Further, NAMPT expression in CD8^+^ T cells is necessary to produce NAD^+^ and induce anti-tumor effects^[103]^. In tumor-infiltrating lymphocytes (TILs), NAMPT and NAD^+^ levels are lower compared to peripheral T cells^[103]^, suggesting that the TIME induces NAD^+^ depletion in TILs, leading to impaired function. Mechanistically, NAD^+^ deficiency in TILs drives mitochondrial dysfunction and reduces ATP production, whereas supplementation with nicotinamide (NAM), the substrate of NAMPT, reverses these effects to promote a strong anti-tumor immune response *in vivo*^[103]^. Interestingly, TCR stimulation in CD8^+^ T cells leads to a 16-fold upregulation of NAMPT, compared to 1.3-fold upregulation in Tregs^[104]^. This suggests that CD8^+^ T cells rely more heavily on NAMPT expression and NAD^+^ levels compared to Tregs, giving these immunosuppressive cells an advantage in the NAD^+^-depleted TIME. Consistently, Tregs are particularly sensitive to NAD^+^-induced cell death^[105]^, and systemic NAD^+^ treatment preferentially depleted Tregs, leading to decreased tumor volume^[106]^. To date, there are several pre-clinical and clinical studies investigating the use of NAMPT inhibitors in both solid and hematologic malignancies^[107]^. However, systemic inhibition of NAMPT might have profound adverse effects on CD8^+^ T cell function, decreasing the drugs’ efficacy. Perhaps these types of drugs are more effective in cancers that do not have high T cell infiltration but overexpress NAMPT.

In immune cells, CD38 is inversely correlated with NAD^+^ levels because it degrades NAD^+^ to NAM and ADP-ribose^[108,109]^. These derivates of NAD^+^ are important secondary messengers that regulate intracellular calcium levels and storage, which in turn mediates T cell differentiation and activation^[109]^. CD38 expression is a marker of T cell exhaustion that contributes to adverse epigenetic modifications in CD8^+^ TILs^[110]^. Further, high expression of CD38, PD-1, and CD101 correlates with the inability of CD8^+^ T cells to undergo epigenetic reprogramming to reverse the exhausted state^[110]^. Conversely, inhibiting CD38 expression in Tregs and B-regulatory cells induced cell death, but drove proliferation of cytotoxic T cells, likely due to depletion of the immunosuppressive populations^[111]^. Consistently, mice deficient in CD38 expression exhibited lower Treg numbers as a result of increased NAD^+^ levels^[106]^. CD38 expression on tumor cells has also been implicated in a variety of solid and hematologic malignancies^[112-116]^. Increased CD38 expression on malignant cells results in acquired resistance to anti-PD-1/PD-L1 therapy by driving CD8^+^ T cells towards an exhausted state^[114]^. Moreover, CD8^+^ T cell function was found to be inhibited by CD38-mediated adenosine production, and anti-PD-L1 and CD38 combination therapy synergistically inhibited the growth of murine lung adenocarcinoma tumors^[114]^. Currently, there are two approved anti-CD38 monoclonal antibody treatments (Daratumumab and Isatuximab) and one in clinical trials (MOR202) to treat multiple myeloma; however, these drugs do not inhibit the ectoenzymatic activity of CD38, rather they induce antibody-dependent cell-mediated cytotoxicity^[117-119]^. There are several drugs in pre-clinical stages that target the ectoenzymatic activity of CD38 to increase NAD^+^ levels for different diseases^[120-122]^. While these drugs are not yet being evaluated in the oncologic space, it would be advantageous because inhibiting CD38 is both beneficial for T cells and detrimental for malignant and immunosuppressive cells, thus eliminating the need for cell-specific drugs.

Taken together, these data demonstrate an important role for lactate, adenosine, and NAD^+^ in regulating immune cell function and ultimately controlling cancer development and progression. Further, pre-clinical studies show promising results that combining these treatments with existing ICB therapies can remodel the TIME to boost the anti-tumor immune response. Thus, continued pre-clinical and clinical efforts are needed to determine whether resistance to anti-PD-1/PD-L1 therapy is ablated when combined with approved anti-CD39/CD73/A2AR/CD38 treatments.

## AMINO ACID METABOLISM

Amino acid metabolism is widely implicated in oncogenesis due to the necessity of amino acids in protein synthesis, epigenetic modifications, and fueling energetic processes. Of the 20 amino acids, only a handful are well-studied in the context of immuno-oncology metabolism and resistance to ICB. Because tryptophan is thoroughly researched in this space and was recently comprehensively reviewed^[123]^, we wanted to focus on amino acids that are sometimes overlooked but still immensely important in regulating cancer development and progression. As such, this section will discuss how tumor-derived alterations in arginine, glutamine, and methionine metabolism contribute to anti-tumor immunity and how modifying the metabolism of these amino acids helps diminish resistance to anti-PD-1/PD-L1 therapy.

### Arginine

**Figure 2:**
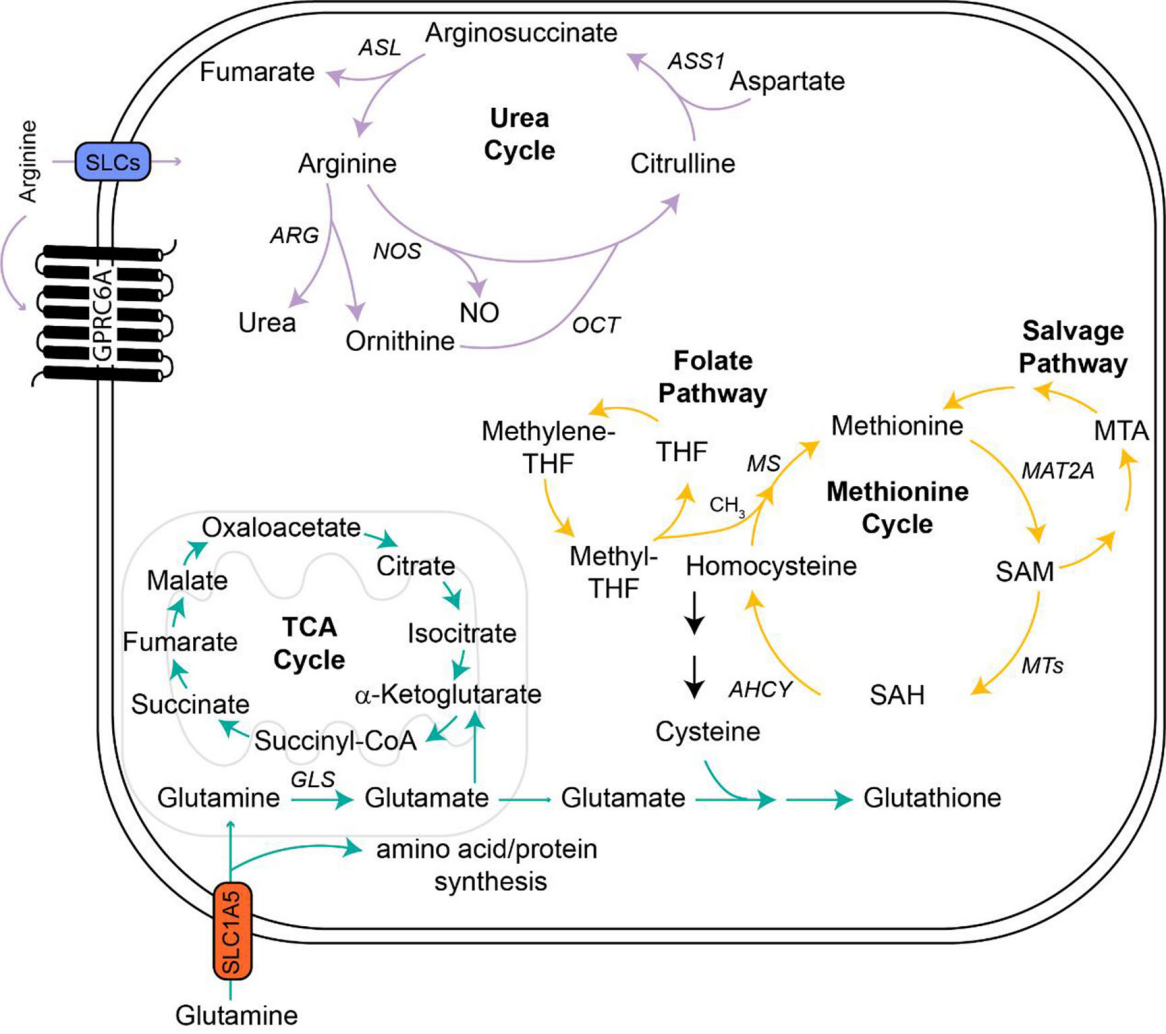
Metabolic pathways of arginine, glutamine, and methionine. Extracellular arginine binds to GPRC6A to drive intracellular arginine signaling or it is imported through various SLC transporters depending on the cell type. Arginine can also be formed through metabolism of citrulline in the urea cycle. Once inside the cell, arginine is catabolized through NOS to form NO or ARG into urea and ornithine, the latter of which is converted back into citrulline to fuel the urea cycle. Glutamine is similarly imported through a variety of SLCs, with SLC1A5 being the predominant transporter on T cells. Intracellular glutamine is used for amino acid/protein synthesis or transported to the mitochondria and converted to glutamate via GLS. In the mitochondria, glutamate is converted to a-Ketoglutarate to fuel the TCA cycle. In the cytosol, glutamate combines with cysteine to form glutathione to combat oxidative stress. Cysteine is generated in part through metabolism of homocysteine in the methionine cycle, which generates methionine for various cellular processes. Methionine is generated by re-methylation of homocysteine through donation of CH_3_ by methyl- THF in the folate cycle. Methionine is then converted to SAM, an indispensable methyl donor, and subsequently SAH following loss of the methyl group. SAM is also involved in the methionine salvage pathway that restores intracellular methionine levels. AHCY: Adenosylhomocysteinase; ARG: arginase; ASL: argininosuccinate lyase; ASS1: argininosuccinate synthase 1; CH_3_: a methyl group; GLS: glutaminase; GPRC6A: G protein-coupled receptor family C group 6 member A; MAT2A: methionine adenosyltransferase 2A; MS: methionine synthase; MTA: 5′-methylthioadenosine; MTs: methyltransferases; NO: nitric oxide; NOS: nitric oxide synthase; OCT: ornithine transcarbamoylase; SAH: S-adenosyl-L-homocysteine; SAM: S-adenosylmethionine; SLC: solute carrier; TCA: tricarboxylic acid; THF: tetrahydrofolate.

Arginine is considered a non-essential amino acid in normal cells because it can be imported or synthesized through citrulline metabolism in the urea cycle^[124]^ [Figure 2]. Conversely, arginine is also catabolized through the urea cycle to form urea and ornithine through arginase (ARG) enzymes^[124]^. Extracellular arginine also participates in the activation of intracellular signaling pathways by binding to G protein-coupled receptor family C group 6 member A (GPRC6A)^[125]^. While arginine itself is important for many cellular processes, it is also a precursor for the synthesis of polyamines, which are organic compounds that facilitate cell proliferation and are upregulated in a variety of cancers^[126-128]^. Similarly, nitric oxide synthase (NOS) metabolizes arginine to nitric oxide (NO), which promotes angiogenesis and metastasis, and dampens the immune response^[129]^.

In malignant cells, arginine helps sustain tumor-promoting functions, and arginine starvation results in detrimental effects, such as ROS formation, mitochondrial dysfunction, and cell death^[130-135]^. Despite this, metabolic rewiring of the urea cycle in tumor cells results in increased ornithine and proline synthesis and decreased arginine synthesis^[131]^. Moreover, many cancer types have decreased expression of arginosuccinate synthase 1 (ASS1), which catalyzes the penultimate step in arginine synthesis^[130]^. As such, arginine is considered an essential amino acid in malignant cells, and they must rely on exogenous uptake to sustain their metabolic demands^[130-132]^. On the other hand, T cells are completely reliant on exogenous arginine because they do not express ASS1^[136,137]^, meaning they must compete with tumor cells and immunosuppressive cells for arginine.

T cell function is highly disrupted by arginine depletion within the TIME, which is mediated by both malignant cells^[138-141]^ and immunosuppressive cells^[142-147]^. In T cells, arginine is important in regulating CD3z expression, which is necessary for proper antigen recognition by the TCR-CD3 complex^[148-151]^. For example, ARG2-dependent depletion of arginine by murine renal cell carcinoma cells leads to decreased expression of CD3z in T cells^[139]^. Sufficient arginine levels are also necessary during T cell activation because arginine is quickly metabolized to fuel downstream processes^[152]^. Moreover, decreased systemic arginine levels in Lewis lung carcinoma^[150]^ and arginine depletion via ARG1 from cancer-derived exosomes in ovarian carcinoma^[153]^ inhibit antigen-specific proliferation of CD8^+^ TILs. Arginine depletion also impairs the effector function of CD8^+^ T cells by preventing the secretion of IFNg and granzyme B^[154,155]^. On the other hand, arginine supplementation in CD8^+^ T cells induces metabolic rewiring from glycolysis towards oxidative phosphorylation to promote proliferation, survival, and anti-tumor responses^[152]^.

Several promising pre-clinical studies have demonstrated that targeting arginine metabolism in combination with anti-PD-1/PD-L1 treatment increases efficacy in overcoming resistance. Employing anti-PD-1 treatment in combination with vaccine inhibition of ARG1 synergistically impaired tumor growth and led to increased CD8^+^ T cell infiltration in mouse models of colorectal carcinoma and fibrosarcoma^[156]^. Further, systemic arginine supplementation with anti-PD-1 or PD-L1 treatment increased CD8^+^ T cell infiltration and exhibited more efficacious results than monotherapy in mouse models of colon carcinoma^[157]^ and osteosarcoma^[158]^. Utilizing a unique approach, researchers engineered an *E. coli* strain that localizes to the TIME and converts ammonia to arginine^[159]^. This innovative method promoted continuous arginine supplementation in murine colorectal carcinoma tumors, leading to increased CD8^+^ T cell infiltration and synergistic anti-tumor effects when combined with anti-PD-L1 treatment^[159]^. Extensive pre-clinical studies for a novel ARG1/2 inhibitor (OATD-02) have shown promising results alone and in combination with both anti-PD-1 and -PD-L1, and researchers are hopeful this drug will enter first-in-human clinical trials soon^[150,160-162]^. Moreover, the ARG1 inhibitor CB-1158 entered first-in-human clinical trials in 2017 and was evaluated with anti-PD-1 treatment^[163-165]^. The results indicate that CB-1158 monotherapy and combination with anti-PD-1 are well-tolerated and elicit a response in solid tumors^[163-165]^.

A considerable amount of evidence demonstrates that tumor-mediated depletion of arginine negatively impacts CD8^+^ T cell function and the anti-tumor response. Additionally, the enhanced anti-tumor effects seen by combining anti-PD-1/PD-L1 with ARG inhibitors or arginine supplementation demonstrate that altering tumor metabolism could have profound effects on the efficacy of ICB. However, continued pre-clinical and clinical efforts are necessary to identify additional ways to target tumor-derived arginine metabolism and reinvigorate the anti-tumor immune response to improve ICB.

### Glutamine

Glutamine has many essential functions, such as supporting the formation of nucleotides and non-essential amino acids, protein synthesis, energy metabolism, and maintaining intracellular redox states^[166]^. Import of glutamine is facilitated by many transporters, predominantly SLC1A5^[136,167]^ [Figure 2]. Once inside the cell, glutamine is transported to the mitochondria to be converted to glutamate via glutaminase enzymes^[166]^. In the cytosol, glutamate serves as a precursor for glutathione synthesis, which is a strong antioxidant^[166]^. The metabolism of glutamine also drives the formation of NADPH, which is critical for restoring the intracellular redox balance by reducing oxidized glutathione^[168]^. In the mitochondria, glutamate is converted to a-Ketoglutarate to drive the TCA cycle^[166]^.

Many cancers exhibit a dependence on or addiction to glutamine. As such, increased glutaminolysis is highly important for ATP production, redox homeostasis, and activation of various oncogenic signaling pathways in tumor cells^[168-170]^. Glutamine fuels KRAS signaling in pancreatic adenocarcinoma^[168]^, mTORC1 signaling in osteosarcoma and cervical cancer cells^[170]^, and promotes lipid biogenesis under hypoxic conditions to provide additional energy sources^[171]^. Hypoxia also drives the mitochondrial import of glutamine to support ATP and glutathione production to combat oxidative stress and promote uncontrolled cell growth^[172]^. Interestingly, data suggest that some cancers will adapt to the glutamine-deprived TIME and will cease to rely on glutamine. In patient-derived melanoma tumors, for example, excess dietary glutamine inhibits cell growth^[173]^.

T cells require glutamine for a variety of functions during differentiation and development^[174]^; thus, there is stiff competition between tumor cells and T cells for glutamine consumption. Ligation of CD3 and CD28 on T cells induces glutamine uptake via ERK and calcineurin pathways to sustain T cell activation, proliferation, and cytokine production^[175,176]^. Interestingly, glutamine is also required for glucose uptake and glycolysis in activated CD8^+^ T cells, and proper effector functions were dependent on both glucose and glutamine^[177]^. As such, increasing glutamine availability for T cells, while depriving tumor cells and immunosuppressive cells, has strong anti-tumor effects. For example, selectively inhibiting glutamine uptake in triple-negative breast cancer cells increased CD8^+^ T cell activation and effector function by promoting glutathione production^[178]^. On the other hand, non-specific intracellular depletion of glutamine leads to impaired mitochondrial function and CD8^+^ T cell apoptosis^[179]^, likely due to increased oxidative damage from reduced glutathione production. Data also suggest the temporal importance of glutamine availability in driving T cell function. During TCR stimulation, glutamine deprivation decreases PD-1 and increases Ki67 expression^[180]^, suggesting that glutamine abundance needs to be tightly regulated at various stages of T cell development to ensure proper functionality. As discussed in previous sections, immunosuppressive cells largely thrive in the nutrient-deprived TIME. Specifically, tumor-associated macrophages respond to low glutamine levels by secreting IL-23 to promote Treg proliferation and activation, resulting in diminished CD8^+^ T cell function^[181]^.

Several reports have demonstrated that inhibiting tumor-associated glutamine metabolism in combination with anti-PD-1/PD-L1 therapies may be a promising approach to restore CD8^+^ T cell function and overcome resistance. Because glutamine deprivation promotes T cell dysfunction, specifically inhibiting glutamine metabolism in tumor cells would yield the most efficacious results. Two separate groups found that glutamine deprivation in cell lines of human clear cell renal carcinoma^[182]^, human non-small cell lung carcinoma^[183]^, and mouse colorectal carcinoma^[183]^ induced PD-L1 expression, which would theoretically boost anti-PD-L1 response. Byun *et al.* found that anti-PD-L1 monotherapy had almost no effect on tumor volume in murine colorectal carcinoma models^[183]^. However, tumor-specific inhibition of glutamine uptake and glutaminase activity in combination with anti-PD-L1 therapy strongly induced CD8^+^ T cell proliferation and granzyme B production, while abating tumor growth^[183]^. Similarly, another group targeted tumor-derived glutamine enzymes by creating a prodrug that is only activated by TIME-restricted enzymes to limit the cytotoxic effects of systemic glutamine antagonism^[184]^. This treatment method decreased glycolysis in malignant cells, decreased hypoxia, acidosis, and nutrient depletion within the TIME, and increased activation of and oxidative phosphorylation in CD8^+^ T cells^[184]^. In combination with anti-PD-1 therapy, tumor-specific glutamine antagonism synergistically reduced tumor growth and increased survival in murine colorectal carcinoma tumors^[184]^. Conversely, employing a non-tumor cell specific glutaminase inhibitor does not yield the same efficacious results. Serine/threonine kinase 11 (STK11) phosphorylates AMPK to regulate a variety of downstream pathways, such as cell growth and proliferation, lipid metabolism, and PD-L1 expression^[185]^. Several studies have shown that STK11 mutations, resulting in loss of function, are associated with resistance to anti-PD-1 treatment^[186-188]^. Building on this, one group found that STK11-mutated lung adenocarcinomas from both patient samples and cancer cell lines exhibited increased glutamate production, so they hypothesized that targeting glutaminase would be a viable way to overcome resistance to anti-PD-1 treatment^[189]^. However, they found that using a non-tumor cell-specific glutaminase inhibitor in combination with anti-PD-1 severely impeded CD8^+^ T cell clonal expansion and anti-tumor functions, and anti-PD-1 efficacy was dependent on intact CD8^+^ T cell glutaminase activity^[189]^.

These data demonstrate a promising future for targeting glutamine metabolism to bolster CD8^+^ T cell effector function and combat ICB resistance. However, it also highlights the importance of finding ways to specifically target malignant cells due to the highly conserved nature of these metabolic pathways.

### Methionine

Methionine is an essential amino acid that is involved in a variety of metabolic pathways, such as methylation reactions, homocysteine synthesis, and the folate pathway [Figure 2]. This metabolite also cooperates with arginine and glutamine to promote polyamine and glutathione synthesis, respectively^[190]^. In the methionine pathway, methionine is converted to S-adenosyl methionine (SAM), which is critical for the methylation of histones, DNA, RNA, proteins, and various metabolites^[191]^. The loss of a methyl group converts SAM to S-adenosyl homocysteine (SAH), and subsequently homocysteine, which is ultimately metabolized to glutathione^[192]^. Methionine regeneration is supported by the metabolism of SAM through the salvage pathway^[192]^ and through the re-methylation of homocysteine via intermediates in the folate pathway^[193]^.

The role of methionine in malignant transformation and growth is not as well-studied as other metabolites, but its wide consumption in cancer cells suggests its importance^[194,195]^. In tumor-initiating cells, exogenous methionine is consumed at extreme rates, leading to pro-tumorigenic epigenetic modifications through methionine adenosyltransferase 2A (MAT2A), which metabolizes methionine to SAM to promote histone methylation^[196]^. In the presence of methionine, malignant cells activate c-MYC, leading to increased MAT2A activity and tumorigenic genome modifications^[197]^. On the other hand, tumor overexpression of nicotinamide N-methyltransferase (NNMT), which converts SAM to NAD^+^ and 1-Methylnicotinamide, leads to increased NAD^+^ levels, hypomethylation, and tumor progression^[198]^, highlighting that altered methionine metabolism can drive oncogenesis in multiple ways.

In T cells, proper metabolic regulation of methionine and its derivatives is necessary for epigenetic reprogramming during activation and differentiation^[199]^, as evidenced by increased expression of methionine transporters during antigen recognition^[175]^. However, dysregulated methionine metabolism by tumor cells alters the abundance of SAM and 5-methylthioadenosine (MTA)^[200]^, both of which drive the methionine salvage pathway^[201]^. Increased abundance of SAM and MTA within the TIME are associated with T cell exhaustion and expression of inhibitory checkpoint markers^[200]^. These two metabolites decrease chromatin accessibility in CD8^+^ T cells for genes involved in TCR signaling, lymphocyte proliferation and differentiation, and increase the accessibility of PD-1^[200]^. Together, these data indicate that tumor-derived alterations in methionine metabolism have a substantial impact on the anti-tumor immune functions of CD8^+^ T cells, but much remains to be discovered.

Despite the limited studies in this field, two recent reports demonstrate that restricting tumor methionine increases CD8^+^ T cell effector functions and overcomes resistance to anti-PD-1/PD-L1 treatment. The first study shows that dietary restriction of methionine reduces SAM levels in murine colorectal carcinoma tumors^[202]^. Mechanistically, SAM controls the expression of immune inhibitory markers PD-L1 and VISTA through m^6^A methylation, whereby the RNA-binding protein YTHDF1 enhances the translation efficiency of RNA containing m^6^A methylation^[202]^. While anti-PD-1 treatment alone in mouse colorectal carcinoma tumors did not significantly alter tumor volume or CD8^+^ T cell infiltration, depletion of YTHDF1 or restricting methionine in the diet synergized with anti-PD-1 treatment to significantly increase survival probability and CD8^+^ T cell infiltration, while decreasing tumor volume^[202]^. Similarly, the second study found that methionine-dependent histone methylation regulates CD8^+^ T cell anti-tumor activities. Methionine deprivation in CD8^+^ T cells resulted in reduced H3K79me2 methylation and subsequent STAT5 expression^[203]^, which is a critical transcription factor that maintains CD8^+^ T cell effector functions^[204]^. *In vitro*, methionine supplementation increased CD8^+^ T cell survival and IFNg and TNFa production, while inhibiting murine melanoma tumor growth^[203]^. The authors also found that SLC43A2 and SLC7A5 import methionine in malignant cells, but T cells are predominantly dependent on SLC7A5^[203]^. As such, genetic ablation of SLC43A2 in mouse melanoma cells restored CD8^+^ T cell polyfunctionality and survival *in vitro*, and decreased tumor growth *in vivo*^[203]^. While anti-PD-1 treatment or pharmacological inhibition of SLC43A2 alone did not elicit significant anti-tumor effects, combination treatment synergistically increased CD8^+^ T cell function and infiltration, and decreased growth of mouse melanoma and ovarian tumors^[203]^. These data demonstrate that resistance to anti-PD-1 treatment can be negated by restricting methionine availability and metabolism in tumors.

Taken together, the studies in this section have undoubtedly established that targeting amino acid metabolism is an efficacious way to improve the response to anti-PD-1/PD-L1 treatment. Targeting these metabolic pathways proves to be challenging because, unlike the immunosuppressive metabolites that have been discussed, amino acids are beneficial for both T cells and tumor cells. Therefore, therapeutic strategies have to promote amino acid supplementation in T cells but restriction in tumor cells, which is no easy feat. Despite these challenges, researchers have made great strides in pre-clinical settings towards identifying how to alter amino acid metabolism in a way that impedes ICB resistance.

## LIPID METABOLISM

The TIME is enriched with various lipid classes^[205-207]^, which is in contrast to other metabolites that are predominantly depleted. Lipids are ubiquitously important for structural support, energy supply, and signaling, making them essential for the malignant properties of tumors and for the proper function of anti-tumor immune cells. Specifically, cholesterol is indispensable for cell membrane integrity and facilitating cell-to-cell and intracellular signaling, while fatty acids (FAs) are the most abundant lipid intermediate, so they are more readily detectable and their role in cancer biology is better understood. Therefore, this section will highlight how tumor-mediated cholesterol and FA dysregulation within the TIME affects CD8^+^ T cell function and anti-PD-1/PD-L1 resistance.

### Cholesterol

**Figure 3:**
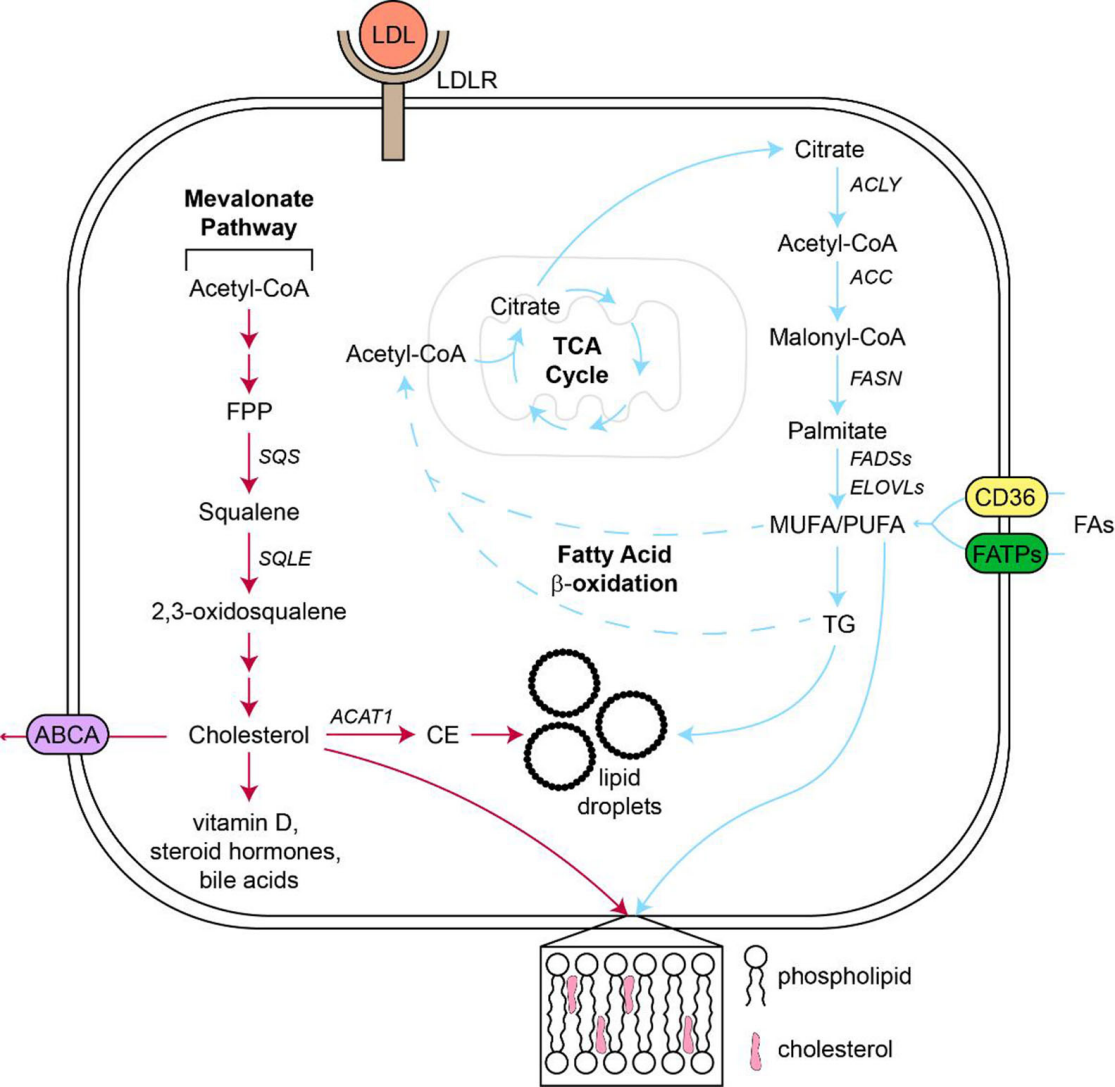
Diagram of cholesterol and FA metabolic pathways. Cholesterol is either imported as LDL through LDLR or it is synthesized through the mevalonate pathway. From there, cholesterol serves as a precursor to vitamin D, steroid hormones, and bile acids or it integrates into the cellular membrane to regulate membrane fluidity and cell signaling. Excess intracellular cholesterol is exported through ABCA or esterified to form CE, which are stored in lipid droplets. FAs are imported via CD36 and fatty acid transport proteins or synthesized through citrate from the TCA cycle. Palmitate, the initial FA that is formed, undergoes elongation and desaturation by ELOVL and FADS enzymes, respectively, to form a variety of FAs with varying chain lengths and degrees of unsaturation. FAs participate in energy metabolism through the FA b-oxidation pathway that generates acetyl-CoA to drive the TCA cycle. Similar to cholesterol, fatty acids are important components of cellular membranes via the formation of phospholipids and excess fatty acids are converted to TG and stored in lipid droplets. ABCA: ATP-binding cassette transporters; ACAT1: Acyl-CoA cholesterol acyl transferase 1; ACC: acetyl-CoA carboxylase; ACLY: ATP citrate lyase; ATP: adenosine triphosphate; CE: cholesteryl esters; ELOVL: elongation of very long chain fatty acids protein; FA: fatty acid; FADS: fatty acid desaturase; FATP: fatty acid transport protein; FASN: fatty acid synthase; FPP: farnesyl diphosphate; LDL: low-density lipoprotein; LDLR: low-density lipoprotein receptor; MUFA: mono-unsaturated fatty acid; PUFA: poly-unsaturated fatty acid; SQS: squalene synthase; SQLE: squalene epoxidase; TCA: tricarboxylic acid; TG: triglyceride.

Cholesterol serves as an important component in cellular membranes and regulates membrane fluidity and cell signaling through the formation of lipid rafts^[208]^ [Figure 3]. Moreover, cholesterol is a precursor for steroid hormones, bile acids, and vitamin D^[208]^. Intracellular cholesterol levels are maintained through biosynthesis via the mevalonate pathway, which converts acetyl-CoA to cholesterol through a series of enzymatic reactions. Additionally, cholesterol is imported as low-density lipoproteins, which are small lipid-enclosed particles that facilitate the systemic transport and cellular import of cholesterol^[209]^. On the other hand, cholesterol is exported through ATP-binding cassette transporters^[210]^. Excess intracellular free cholesterol is converted to cholesteryl esters and stored in lipid droplets, which promote oncogenic signaling and cancer growth^[211]^.

Malignant cells utilize excess cholesterol to sustain their rapid growth and proliferation^[212-214]^ and elevated intracellular cholesterol levels are maintained by increasing import and synthesis and decreasing export^[215,216]^. Altered cholesterol content in malignant cell membranes regulates apoptosis^[217]^, proliferation, metastasis^[218]^, and killing by cytotoxic T cells^[219]^. Cholesterol and its derivatives are also involved in various oncogenic signaling pathways and protein modifications^[220]^. Unsurprisingly, these metabolites are sequestered by tumor cells to promote malignant growth, and dysregulation of cholesterol in the TIME by tumor cells affects the cytotoxic functions of CD8^+^ T cells.

There are multiple ways in which tumor cells directly alter cholesterol metabolism within the TIME to inhibit CD8^+^ T cell function. Protein convertase subtilisin/kexin type 9 (PCSK9) is a secreted enzyme that regulates cholesterol levels by facilitating the degradation of low-density lipoprotein receptors (LDLR)^[221-224]^, which imports low-density lipoprotein cholesterol. Tumor-secreted PCSK9 promotes intratumoral accumulation of cholesterol^[225]^, prevents LDLR and TCR recycling in CD8^+^ TILs^[226]^, and inhibits MCH-1 recycling on tumor cells^[227]^, leading to immune evasion in multiple ways. Further, several reports demonstrate that intratumoral cholesterol accumulation promotes PD-L1 expression^[228-231]^, thereby contributing to immune evasion. Mechanistically, cholesterol binds to the transmembrane domain of PD-L1 to stabilize cell surface expression^[231]^. Cholesterol-derived metabolites produced by malignant cells also dictate anti-tumor response. For example, cholesterol sulfate creates a chemical barrier within the TIME to prevent CD8^+^ T cell infiltration^[232]^. Moreover, cholesterol sulfate-producing tumors are more resistant to ICB therapy^[232]^ than tumors that do not produce this metabolite, demonstrating that targeting tumor-intrinsic cholesterol metabolism could enhance ICB outcomes.

In addition to cholesterol biochemical pathways regulating CD8^+^ T cell function, mechanical forces driven by altered cholesterol levels within tumor cells also influence anti-tumor immune response. Cancer cells accumulate cholesterol within the cell membrane, leading to increased membrane fluidity, or “cell softening”^[219]^. This phenomenon is associated with cancer development and progression because cancer cell softening impairs the cytotoxic effects of T cells, leading to immune escape^[219]^. By reversing these effects and promoting cancer cell stiffening, increased T cell forces and actin accumulation at the immunological synapse enhance tumor killing^[219]^. Notably, cancer cell stiffening did not alter TCR signaling or cytokine production, demonstrating that these effects were purely through mechanical forces^[219]^.

In T cells, maintaining a proper balance between membrane and intracellular cholesterol levels is important for development, activation, and effector functions. Cholesterol in the cell membrane is essential for the intricate formation of lipid rafts which regulate TCR signaling^[233]^. In TILs, several studies report that the allocation of cholesterol towards cell membrane formation instead of storage as cholesterol esters promotes anti-tumor activities. Pharmacologic inhibition in tumor cells and CD8^+^ T cells of acyl-CoA cholesterol acyltransferase 1 (ACAT1), which promotes cholesterol esterification, inhibits cancer cell growth^[234]^. Similarly, another group found that RORa, a nuclear hormone receptor, promotes CD8^+^ T cell membrane cholesterol accumulation by inhibiting cholesterol esterification, thus enhancing anti-tumor functions^[235]^. On the other hand, intracellular cholesterol accumulation in CD8^+^ T cells due to cholesterol enrichment in the TIME leads to endoplasmic reticulum (ER) stress, which causes T cell exhaustion and increased expression of immune checkpoint markers^[207]^. Mechanistically, ER stress promotes upregulation of the ER stress sensing protein XBP1, which drives the expression of immune inhibitory markers, namely PD-1 and 2B4^[207]^. As a result, inhibiting XBP1 or reducing cholesterol in CD8^+^ T cells or the TIME boosts the anti-tumor functions of CD8^+^ T cells^[207]^. These studies demonstrate that shifting cholesterol away from intracellular stores towards membrane formation in T cells might be an effective therapeutic strategy to diminish resistance to ICB therapy.

Given the profound effect of tumor-derived cholesterol on CD8^+^ T cell function, it is no surprise that targeting this altered metabolic pathway inhibits resistance to anti-PD-1 treatment. Building on the idea that allocating cholesterol towards cellular membranes in CD8^+^ T cells is beneficial for the anti-tumor response, researchers found that pharmacologic inhibition of ACAT1 in combination with anti-PD-1 treatment synergistically reduced the growth of mouse melanoma tumors^[236]^. Further, slight anti-tumor effects were observed in four mouse tumor models following genetic ablation of PCSK9, but combination of genetic or pharmacologic inhibition of PCSK9 with anti-PD-1 resulted in robust synergistic effects to increase MHC-I expression and survival and reduce growth of murine melanoma and colorectal carcinoma tumors^[227]^. Another emerging target is squalene epoxidase (SQLE), which catalyzes one of the rate-limiting steps in sterol synthesis [Figure 3]. Bioinformatics approaches have identified a negative correlation between SQLE expression in human pancreatic adenocarcinoma and immune cell infiltration and immunotherapy response^[237]^, prompting the need for further validation of this potential target. While the intersection of tumor-mediated cholesterol metabolism and ICB response is not as robust as other metabolic programs, these recent studies hint at how this relationship can be exploited to overcome ICB resistance.

### Fatty acids

Similar to cholesterol, FAs have a variety of cellular functions, including cell membrane formation through phospholipids, energy metabolism, and precursors for signaling lipids [Figure 3]. Intracellular FA abundance is regulated by import through CD36 or FA transport proteins and synthesis via fatty acid synthase (FASN) from acetyl-CoA or malonyl-CoA^[238]^. FAs undergo modifications to chain length to form long-chain FAs (LCFAs) or very long-chain FAs (VLCFAs) and saturation to form mono-, di-, and poly-unsaturated FAs. Saturation and chain length dictate FA function and their role in oncogenesis^[239]^. In energy metabolism, FAs are subject to fatty acid b-oxidation (FAO) in the mitochondria to generate FADH, NADH, and acetyl-CoA to fuel a variety of energetic processes^[240]^.

The increased demand for FAs in malignant cells sustains their rapid proliferation by serving as an energy source via FAO and as an indispensable component for cell membrane formation. Moreover, certain FAs are important precursors for a variety of oncogenic signaling mediators^[241-243]^. To meet these metabolic demands, cancer cells will increase the uptake and synthesis of fatty acids, while also inducing lipolysis of neighboring adipocytes^[244-248]^. Continuous evidence is emerging that altered FA metabolism by tumor cells alters the lipidome in the TIME, contributing to CD8^+^ T cell dysfunction. However, the effect of tumor-derived FA metabolic alterations on ICB resistance is not well-studied.

Malignant cells exploit the increased lipid availability in patients with obesity and remodel the TIME to inhibit CD8^+^ T cell function and promote cancer growth. High-fat diet-induced obesity in multiple mouse models of cancer alters the metabolic profile of malignant cells to increase FA uptake and utilization and creates an immunosuppressive TIME that inhibits CD8^+^ T cell infiltration and function^[249]^. Moreover, inhibiting obesity-induced metabolic rewiring in murine colorectal carcinoma tumors restores CD8^+^ TIL function and increases anti-tumor immune function^[249]^. Mechanistically, researchers found that CD8^+^ T cells in obesity-associated breast cancer tumors exhibit ligation of leptin and PD-1 to reduce effector functions through activation of STAT3, which promotes FAO and inhibits glycolysis^[250]^. PD-1 ligation also promotes FAO in T cells through upregulation of carnitine palmitoyltransferase 1A (CPT1A), an essential enzyme involved in FAO^[251]^. Further, obesity in mice, humans, and non-human primates leads to increased PD-1 expression and CD8^+^ T cell exhaustion^[252]^. These data are consistent with the notion that CD8^+^ T cells exhibit a shift from glycolysis to FAO as they become exhausted, highlighting the need to further explore targeting metabolic reprogramming as a way to reinvigorate CD8^+^ T cells and abate ICB resistance.

Similar to obese models of cancer, non-obese models show that CD8^+^ T cell function is inhibited by an overabundance of FAs within the TIME. In response to excess lipid content within the TIME, CD8^+^ TILs exhibit increased intracellular lipid levels compared to peripheral CD8^+^ T cells^[205]^. Exhaustion in CD8^+^ TILs is characterized by the expression of CD36, which imports oxidized low-density lipoproteins, oxidized phospholipids, and long-chain fatty acids^[205]^. Increased uptake of oxidized low-density lipoproteins promotes lipid peroxidation in CD8^+^ TILs, leading to decreased cytokine production and effector function^[205]^. Moreover, the accumulation of VLCFAs within the TIME drives the uptake of LCFAs in CD8^+^ T cells, and instead of serving as an energy source, they promote mitochondrial dysfunction, lipotoxicity, and exhaustion^[253]^. Like cancer cells, immunosuppressive cells, such as Tregs, macrophages, and MDSCs, rely heavily on exogenous FAs to sustain their increased rate of FAO^[254-257]^. In this regard, increased FA abundance within the TIME hinders CD8^+^ T cell function, while benefiting malignant and immunosuppressive cells.

FAs are the building blocks for a variety of bioactive lipids, which are involved in signaling pathways. Tumor cells, and to a lesser extent CAFs^[258]^, secrete the enzyme autotaxin (ATX) that converts ubiquitously available lysophosphatidylcholine (LPC) to the bioactive lipid lysophosphatidic acid (LPA)^[259]^. LPA modulates numerous signaling pathways through lysophosphatidic acid receptors 1-6 (LPAR1-6), which are present on a variety of cell types^[259]^. In malignant cells, the ATX/LPA axis also functions in an autocrine manner by promoting oncogenic signaling through LPAR1^[260]^. On CD8^+^ T cells, tumor-derived LPA binds to LPAR6 and prevents tumor infiltration by inhibiting migration^[260]^. LPA also signals through LPAR5 on CD8^+^ T cells to induce cytoskeletal dysfunction, immunological synapse malformation, and impaired cytokine secretion and intracellular calcium release^[261-263]^. LPAR5 signaling on CD8^+^ T cells also induces an exhausted-like state by promoting metabolic stress through ROS production and ultimately impairing antigen-specific killing^[264]^. The recent development of a first-in-class ATX inhibitor demonstrated tumor growth inhibition in mouse models of breast cancer^[265,266]^. The safety of this compound was tested in Phase I clinical trials in 2021, where the drug was well-tolerated with no significant clinically adverse effects^[266]^. These promising results demonstrate the previously unexplored capacity to target ATX in solid tumors, with the future potential to combine this treatment with pre-existing ICB therapies.

There is very limited research on targeting FA metabolism in combination with anti-PD-1/PD-L1 therapy, but more evidence is emerging that supports this approach to overcome ICB resistance. Bioinformatics methods have identified that FASN expression in patients with bladder cancer, melanoma, and non-small cell lung carcinoma is linked to immune infiltration and ICB response^[267,268]^. Interestingly, ICB is more efficacious in obese patients with melanoma compared to non-obese patients^[252,269-272]^. While this may seem contradictory, obesity drives PD-1 expression on CD8^+^ T cells, thus eliciting a more robust response. On the other hand, CD8^+^ TILs in pancreatic adenocarcinoma exhibit increased expression of checkpoint inhibitors, but ICB therapy largely fails^[273-275]^. The variability in ICB response between cancer types prompts the need for a deeper understanding of the mechanisms that contribute to resistance. To further complicate things, under hypoxic and hypoglycemic conditions, pharmacologically enhancing FA catabolism in CD8^+^ T cells promotes effector function^[206]^. Moreover, anti-PD-1 treatment, in combination with increased FA catabolism, synergistically reduced the volume of murine melanoma tumors and promoted anti-tumorigenic metabolic reprogramming in CD8^+^ T cells^[206]^. These data suggest that under stressful conditions, i.e., oxygen and glucose depletion, increased FAO is required for CD8^+^ T cell function, but this contradicts other studies that demonstrate a shift towards FAO promotes exhaustion.

Together, these research efforts have laid the groundwork to further characterize the intricate relationship between tumor-mediated cholesterol and FA metabolism and CD8^+^ T cell function within the TIME. To date, it is not clear whether inhibiting cholesterol or FA metabolism is a viable treatment option to improve response to anti-PD-1/PD-L1 therapies. As new data emerges, researchers will have a better understanding of the tumor-specific cholesterol and FA metabolic programs that are exploited by cancer cells and if these can be targeted to prevent ICB resistance.

## CONCLUSION

**Figure 4:**
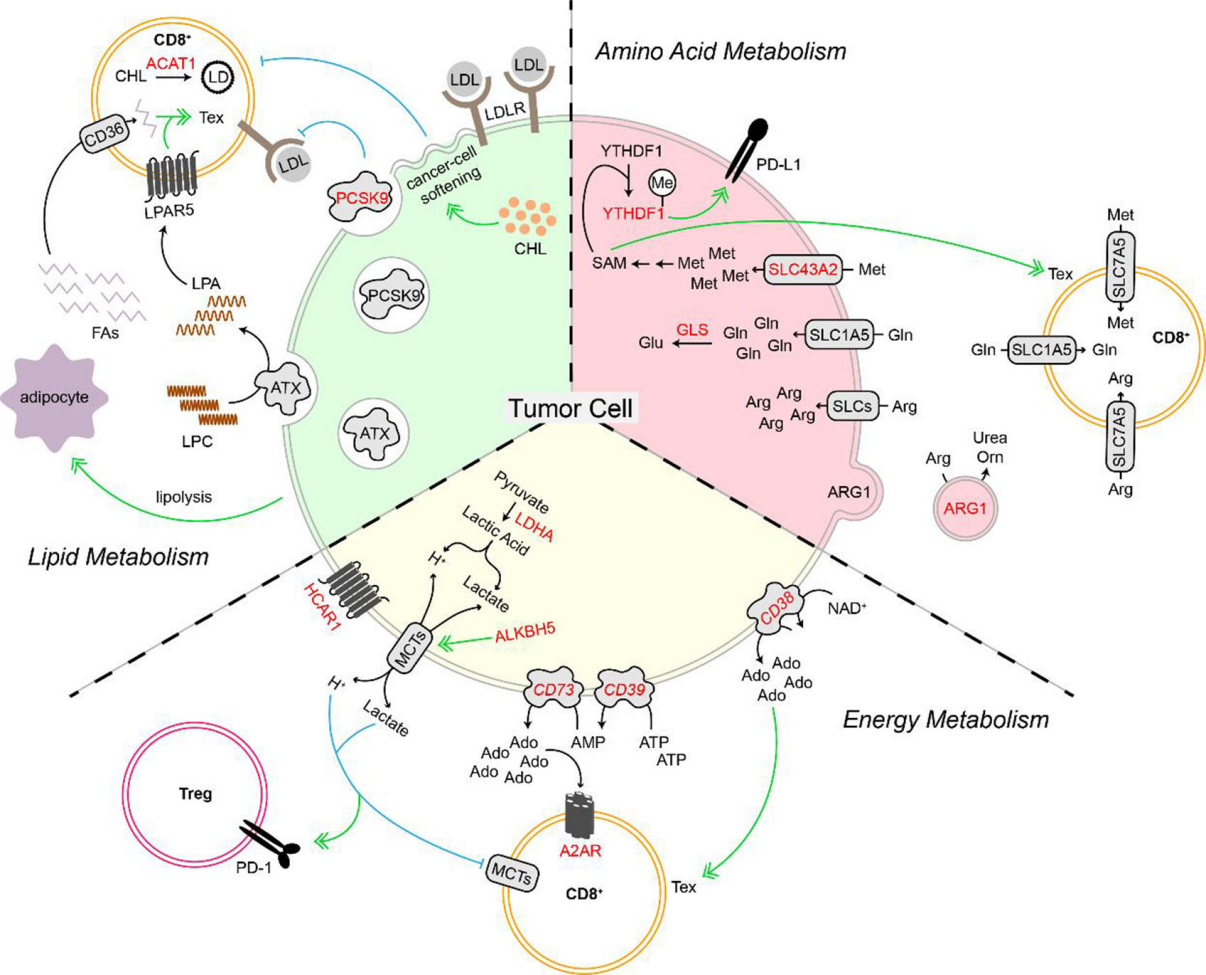
Summary schematic of how altered tumor-intrinsic energy, amino acid, and lipid metabolism drive CD8^+^ T cell dysfunction and resistance to anti-PD-1/PD-L1 treatment. Targets in red are described in the previous sections and modulating these targets overcomes resistance to anti-PD-1/PD-L1 therapy. ACAT1: Acyl-CoA cholesterol acyl transferase 1; Ado: adenosine; ALKBH5: alkB homolog 5, RNA demethylase; Arg: arginine; ARG1: arginase 1; ATX: autotaxin; A2AR: adenosine A2A receptor; CD8^+^: CD^+^ T cell; CHL: cholesterol; FAs: fatty acids; Gln: glutamine; GLS: glutaminase; Glu: glutamate; HCAR1: hydroxycarboxylic acid receptor 1; LD: lipid droplet; LDHA: lactate dehydrogenase A; LDL: low-density lipoprotein; LDLR: low-density lipoprotein receptor; LPA: lysophosphatidic acid; LPAR5: lysophosphatidic acid receptor 5; LPC: lysophosphatidylcholine; MCT: monocarboxylate transporter; Me: methyl; Met: methionine; NAD^+^: nicotinamide adenine dinucleotide; Orn: ornithine; PCSK9: proprotein convertase subtilisin/kexin type 9; PD-L1: programmed cell death ligand 1; PD-1: programmed cell death protein 1; SAM: S-adenosylmethionine; SLC: solute carrier; Tex: CD8^+^ T cell exhaustion; Treg: T regulatory cell; YTHDF1: YTH N6-methyladenosine RNA binding protein F1.

While ICB therapies have been an imperative advancement in cancer treatment, a majority of patients exhibit resistance, prompting the need for researchers to identify and target these resistance mechanisms. This review has provided a multitude of examples wherein tumor-intrinsic alterations to energy, amino acid, and lipid metabolism have a significant impact on CD8^+^ T cell function and resistance to anti-PD-1/PD-L1 therapies [Table 1 and Figure 4]. In many of the studies presented here, anti-PD-1/PD-L1 therapy alone elicits limited anti-tumor effects but, when combined with targeting metabolic pathways, the response is significantly more robust. Nevertheless, there are a limited number of metabolism-targeting drugs that make it to the clinic because these pathways are highly conserved and not tumor-cell specific. As such, this warrants either unique ways to mitigate systemic effects, some of which have been provided in this review, or continued efforts to identify tumor-specific pathways. However, the extreme heterogeneity of the TIME, metabolome, and lipidome between cancer types necessitates large research efforts to uncover these distinct metabolic programs.

**Table 1:** Tumor-intrinsic metabolic targets, the resulting metabolites, and the drug or compound used against the target that have been evaluated pre-clinically and/or clinically in combination with anti-PD-1/PD-L1 therapy

**Target (metabolite)**	**Drug/Compound**	**Pre-clinical or clinical**	**Combination with anti-PD-1/PD-L1**	**Ref.**
ALKBH5 (lactate)	ALK-04	Pre-clinical	Anti-PD-1	[42]
LDHA (lactate)	GSK2837808A	Pre-clinical	Anti-PD-1	[43]
HCAR1 (lactate)	3-OBA	Pre-clinical	Anti-PD-1	[44]
A2AR (adenosine)	CPI-444	Clinical	Anti-PD-L1	[84]
A2AR (adenosine)	DZD2269	Pre-clinical	Anti-PD-1	[85]
CD39 (adenosine)	IPH5201	Clinical	Anti-PD-L1	[89]
CD39 (adenosine)	IPH5201	Pre-clinical	Anti-PD-L1	[88-90]
CD39 (adenosine)	TTX-030	Clinical	Anti-PD-1	[91]
CD73 (adenosine)	MEDI9447 (oleclumab)	Clinical	Anti-PD-L1	[92,93]
A2AR (adenosine)	Nanoreactor	Pre-clinical	Anti-PD-1	[94]
CD39 (adenosine)	POM-1	Pre-clinical	Anti-PD-1	[95]
CD39 (adenosine)	ARL67156	Pre-clinical	Anti-PD-1	[96]
CD38 (NAD^+^)	Anti-CD38 and RHein	Pre-clinical	Anti-PD-L1	[114]
ARG1 (arginine)	Vaccine	Pre-clinical	Anti-PD-1	[156]
ARG1/2 (arginine)	OATD-02	Pre-clinical	Anti-PD-1	[150,160-162]
ARG (arginine)	CB-1158	Clinical	Anti-PD-1	[163-165]
SLC1A5 (glutamine)	V-9302	Pre-clinical	Anti-PD-L1	[183]
Glutamine-utilizing enzymes (glutamine)	JHU083	Pre-clinical	Anti-PD-1	[184]
YTHDF1 (methionine)	Short-hairpin knockdown of YTHDF1	Pre-clinical	Anti-PD-L1	[202]
SLC43A2 (methionine)	BCH	Pre-clinical	Anti-PD-L1	[203]
ACAT1 (cholesterol)	CI-1011	Pre-clinical	Anti-PD-1	[236]
PCSK9 (cholesterol)	AMG-145 and D10335	Pre-clinical	Anti-PD-1	[227]

ACAT1: Acyl-CoA cholesterol acyl transferase 1; ALKBH5: alkB homolog 5; ARG1: arginase 1; A2AR: adenosine A2A receptor; HCAR1: hydroxycarboxylic acid receptor 1; LDHA: lactate dehydrogenase A; PCSK9: proprotein convertase subtilisin/kexin type 9; PD-L1: programmed cell death ligand 1; PD-1: programmed cell death protein 1; SLC: solute carrier; YTHDF1: YTH N6-methyladenosine RNA binding protein F1.

Future directions for the fields of immuno- and onco-metabolism are rooted in the utilization of metabolomic and lipidomic analyses to understand the metabolic landscape of cancer and develop efficacious cancer treatments. Taking a true multi-omics approach by incorporating proteomics, transcriptomics/spatial transcriptomics, and metabolomics/spatial metabolomics will greatly advance our understanding of targetable pathways, both within malignant cells and T cells. These methods are gaining more traction within the oncology research space and hopefully will be more widely utilized in the coming years.

## PERSPECTIVES

In recent years, immense strides have been made in studying the intersection of metabolism, cancer, and the immune system. In addition to the metabolites and pathways covered in this review, there are a plethora of others waiting to be linked to CD8^+^ T cell dysfunction and ICB resistance. For example, other amino acids and lipid classes, metabolites produced by the gut microbiome, and a closer look at the metabolites associated with oxidative phosphorylation and ATP production. Moreover, there is much to uncover about how tumor-derived metabolic alterations affect other immune and non-immune cell types. Continued research efforts in this field will provide a more comprehensive understanding of tumor-intrinsic metabolic alterations and reveal nuanced ways to target tumor metabolism and overcome resistance to ICB therapies.

## DECLARATIONS

### Copyright

© The Author(s) 2023.

## References

[1] Pardoll DM (2012). The blockade of immune checkpoints in cancer immunotherapy. Nat Rev Cancer.

[2] Thommen DS, Schumacher TN (2018). T cell dysfunction in cancer. Cancer Cell.

[3] Chen L, Han X (2015). Anti-PD-1/PD-L1 therapy of human cancer: past, present, and future. J Clin Invest.

[4] Morad G, Helmink BA, Sharma P, Wargo JA (2021). Hallmarks of response, resistance, and toxicity to immune checkpoint blockade. Cell.

[5] Warburg O, Wind F, Negelein E (1927). The metabolism of tumors in the body. J Gen Physiol.

[6] Hanahan D (2022). Hallmarks of cancer: new dimensions. Cancer Discov.

[7] Pavlova NN, Zhu J, Thompson CB (2022). The hallmarks of cancer metabolism: still emerging. Cell Metab.

[8] Leone RD, Powell JD (2020). Metabolism of immune cells in cancer. Nat Rev Cancer.

[9] Binnewies M, Roberts EW, Kersten K (2018). Understanding the tumor immune microenvironment (TIME) for effective therapy. Nat Med.

[10] Roy DG, Kaymak I, Williams KS, Ma EH, Jones RG (2021). Immunometabolism in the tumor microenvironment. Annu Rev Cancer Biol.

[11] Brown TP, Ganapathy V (2020). Lactate/GPR81 signaling and proton motive force in cancer: role in angiogenesis, immune escape, nutrition, and Warburg phenomenon. Pharmacol Ther.

[12] Halestrap AP, Price NT (1999). The proton-linked monocarboxylate transporter (MCT) family: structure, function and regulation. Biochem J.

[13] Halestrap AP (2013). The SLC16 gene family - structure, role and regulation in health and disease. Mol Aspects Med.

[14] Sandforth L, Ammar N, Dinges LA (2020). Impact of the monocarboxylate transporter-1 (MCT1)-mediated cellular import of lactate on stemness properties of human pancreatic adenocarcinoma cells †. Cancers.

[15] Longhitano L, Forte S, Orlando L (2022). The crosstalk between GPR81/IGFBP6 promotes breast cancer progression by modulating lactate metabolism and oxidative stress. Antioxidants.

[16] Boedtkjer E, Pedersen SF (2020). The acidic tumor microenvironment as a driver of cancer. Annu Rev Physiol.

[17] Notarangelo G, Spinelli JB, Perez EM (2022). Oncometabolite _D_-2HG alters T cell metabolism to impair CD8^+^ T cell function. Science.

[18] Hermans D, Gautam S, García-Cañaveras JC (2020). Lactate dehydrogenase inhibition synergizes with IL-21 to promote CD8^+^ T cell stemness and antitumor immunity. Proc Natl Acad Sci U S A.

[19] Rostamian H, Khakpoor-Koosheh M, Jafarzadeh L (2022). Restricting tumor lactic acid metabolism using dichloroacetate improves T cell functions. BMC Cancer.

[20] Franchina DG, Dostert C, Brenner D (2018). Reactive oxygen species: involvement in T cell signaling and metabolism. Trends Immunol.

[21] Fischer K, Hoffmann P, Voelkl S (2007). Inhibitory effect of tumor cell-derived lactic acid on human T cells. Blood.

[22] Brand A, Singer K, Koehl GE (2016). LDHA-associated lactic acid production blunts tumor immunosurveillance by T and NK cells. Cell Metab.

[23] Macian F (2005). NFAT proteins: key regulators of T-cell development and function. Nat Rev Immunol.

[24] Ping W, Senyan H, Li G, Yan C, Long L (2018). Increased lactate in gastric cancer tumor-infiltrating lymphocytes is related to impaired T cell function due to miR-34a deregulated lactate dehydrogenase A. Cell Physiol Biochem.

[25] Pauken KE, Wherry EJ (2015). Overcoming T cell exhaustion in infection and cancer. Trends Immunol.

[26] Kaymak I, Luda KM, Duimstra LR (2022). Carbon source availability drives nutrient utilization in CD8^+^ T cells. Cell Metab.

[27] Hui S, Ghergurovich JM, Morscher RJ (2017). Glucose feeds the TCA cycle via circulating lactate. Nature.

[28] Faubert B, Li KY, Cai L (2017). Lactate metabolism in human lung tumors. Cell.

[29] Quinn WJ 3rd, Jiao J, TeSlaa T (2020). Lactate limits T cell proliferation via the NAD(H) redox state. Cell Rep.

[30] Xia H, Wang W, Crespo J (2017). Suppression of FIP200 and autophagy by tumor-derived lactate promotes naïve T cell apoptosis and affects tumor immunity. Sci Immunol.

[31] Elia I, Rowe JH, Johnson S (2022). Tumor cells dictate anti-tumor immune responses by altering pyruvate utilization and succinate signaling in CD8^+^ T cells. Cell Metab.

[32] Angelin A, Gil-de-Gómez L, Dahiya S (2017). Foxp3 reprograms T cell metabolism to function in low-glucose, high-lactate environments. Cell Metab.

[33] Watson MJ, Vignali PDA, Mullett SJ (2021). Metabolic support of tumour-infiltrating regulatory T cells by lactic acid. Nature.

[34] Stone SC, Rossetti RAM, Alvarez KLF (2019). Lactate secreted by cervical cancer cells modulates macrophage phenotype. J Leukoc Biol.

[35] Kelderman S, Heemskerk B, van Tinteren H (2014). Lactate dehydrogenase as a selection criterion for ipilimumab treatment in metastatic melanoma. Cancer Immunol Immunother.

[36] Nosrati A, Tsai KK, Goldinger SM (2017). Evaluation of clinicopathological factors in PD-1 response: derivation and validation of a prediction scale for response to PD-1 monotherapy. Br J Cancer.

[37] Zhang Z, Li Y, Yan X (2019). Pretreatment lactate dehydrogenase may predict outcome of advanced non small-cell lung cancer patients treated with immune checkpoint inhibitors: a meta-analysis. Cancer Med.

[38] Schouwenburg MG, Suijkerbuijk KPM, Koornstra RHT (2019). Switching to immune checkpoint inhibitors upon response to targeted therapy; the road to long-term survival in advanced melanoma patients with highly elevated serum LDH?. Cancer.

[39] Wang X, Zhang B, Chen X (2019). Lactate dehydrogenase and baseline markers associated with clinical outcomes of advanced esophageal squamous cell carcinoma patients treated with camrelizumab (SHR-1210), a novel anti-PD-1 antibody. Thorac Cancer.

[40] Yin TT, Huang MX, Wang F (2022). Lactate score predicts survival, immune cell infiltration and response to immunotherapy in breast cancer. Front Genet.

[41] Renner K, Bruss C, Schnell A (2019). Restricting glycolysis preserves T cell effector functions and augments checkpoint therapy. Cell Rep.

[42] Li N, Kang Y, Wang L (2020). ALKBH5 regulates anti-PD-1 therapy response by modulating lactate and suppressive immune cell accumulation in tumor microenvironment. Proc Natl Acad Sci U S A.

[43] Kumagai S, Koyama S, Itahashi K (2022). Lactic acid promotes PD-1 expression in regulatory T cells in highly glycolytic tumor microenvironments. Cancer Cell.

[44] Stransky N, Huber SM (2022). Comment on Chen et al. Dual blockade of lactate/GPR81 and PD-1/PD-L1 pathways enhances the anti-tumor effects of metformin. *Biomolecules* 2021, *11*, 1373. Biomolecules.

[45] Feng Q, Liu Z, Yu X (2022). Lactate increases stemness of CD8  + T cells to augment anti-tumor immunity. Nat Commun.

[46] Kaczmarek E, Koziak K, Sévigny J (1996). Identification and characterization of CD39/vascular ATP diphosphohydrolase. J Biol Chem.

[47] Zimmermann H (1992). 5'-nucleotidase: molecular structure and functional aspects. Biochem J.

[48] Horenstein AL, Chillemi A, Zaccarello G (2013). A CD38/CD203a/CD73 ectoenzymatic pathway independent of CD39 drives a novel adenosinergic loop in human T lymphocytes. Oncoimmunology.

[49] Campos-Contreras ADR, Díaz-Muñoz M, Vázquez-Cuevas FG (2020). Purinergic signaling in the hallmarks of cancer. Cells.

[50] Allard B, Longhi MS, Robson SC, Stagg J (2017). The ectonucleotidases CD39 and CD73: novel checkpoint inhibitor targets. Immunol Rev.

[51] Ferretti E, Horenstein AL, Canzonetta C, Costa F, Morandi F (2019). Canonical and non-canonical adenosinergic pathways. Immunol Lett.

[52] Allard B, Allard D, Buisseret L, Stagg J (2020). Publisher correction: the adenosine pathway in immuno-oncology. Nat Rev Clin Oncol.

[53] (2017). Virgilio F, Adinolfi E. Extracellular purines, purinergic receptors and tumor growth. Oncogene.

[54] Pellegatti P, Raffaghello L, Bianchi G, Piccardi F, Pistoia V, Di Virgilio F (2008). Increased level of extracellular ATP at tumor sites: *in vivo* imaging with plasma membrane luciferase. PLoS One.

[55] Mora-García ML, Ávila-Ibarra LR, García-Rocha R (2017). Cervical cancer cells suppress effector functions of cytotoxic T cells through the adenosinergic pathway. Cell Immunol.

[56] Sundström P, Stenstad H, Langenes V (2016). Regulatory T cells from colon cancer patients inhibit effector T-cell migration through an adenosine-dependent mechanism. Cancer Immunol Res.

[57] Shi L, Feng M, Du S (2019). Adenosine generated by regulatory T Cells induces CD8^+^ T cell exhaustion in gastric cancer through A2aR pathway. Biomed Res Int.

[58] Maj T, Wang W, Crespo J (2017). Oxidative stress controls regulatory T cell apoptosis and suppressor activity and PD-L1-blockade resistance in tumor. Nat Immunol.

[59] Giatromanolaki A, Kouroupi M, Pouliliou S (2020). Ectonucleotidase CD73 and CD39 expression in non-small cell lung cancer relates to hypoxia and immunosuppressive pathways. Life Sci.

[60] Vignali PDA, DePeaux K, Watson MJ (2023). Hypoxia drives CD39-dependent suppressor function in exhausted T cells to limit antitumor immunity. Nat Immunol.

[61] Ohta A, Kini R, Ohta A, Subramanian M, Madasu M, Sitkovsky M (2012). The development and immunosuppressive functions of CD4^+^ CD25^+^ FoxP3^+^ regulatory T cells are under influence of the adenosine-A2A adenosine receptor pathway. Front Immunol.

[62] Torres-Pineda DB, Mora-García ML, García-Rocha R (2020). Adenosine augments the production of IL-10 in cervical cancer cells through interaction with the A_2B_ adenosine receptor, resulting in protection against the activity of cytotoxic T cells. Cytokine.

[63] King RJ, Shukla SK, He C (2022). CD73 induces GM-CSF/MDSC-mediated suppression of T cells to accelerate pancreatic cancer pathogenesis. Oncogene.

[64] Ludwig N, Yerneni SS, Azambuja JH (2020). Tumor-derived exosomes promote angiogenesis via adenosine A_2B_ receptor signaling. Angiogenesis.

[65] Ploeg EM, Ke X, Britsch I (2021). Bispecific antibody CD73xEpCAM selectively inhibits the adenosine-mediated immunosuppressive activity of carcinoma-derived extracellular vesicles. Cancer Lett.

[66] Morandi F, Marimpietri D, Horenstein AL, Corrias MV, Malavasi F (2019). Microvesicles expressing adenosinergic ectoenzymes and their potential role in modulating bone marrow infiltration by neuroblastoma cells. Oncoimmunology.

[67] Clayton A, Al-Taei S, Webber J, Mason MD, Tabi Z (2011). Cancer exosomes express CD39 and CD73, which suppress T cells through adenosine production. J Immunol.

[68] Chimote AA, Balajthy A, Arnold MJ (2018). A defect in KCa3.1 channel activity limits the ability of CD8^+^ T cells from cancer patients to infiltrate an adenosine-rich microenvironment. Sci Signal.

[69] Chimote AA, Hajdu P, Kucher V (2013). Selective inhibition of KCa3.1 channels mediates adenosine regulation of the motility of human T cells. J Immunol.

[70] Feske S, Skolnik EY, Prakriya M (2012). Ion channels and transporters in lymphocyte function and immunity. Nat Rev Immunol.

[71] Newton HS, Gawali VS, Chimote AA (2020). PD1 blockade enhances K^+^ channel activity, Ca^2+^ signaling, and migratory ability in cytotoxic T lymphocytes of patients with head and neck cancer. J Immunother Cancer.

[72] Deaglio S, Dwyer KM, Gao W (2007). Adenosine generation catalyzed by CD39 and CD73 expressed on regulatory T cells mediates immune suppression. J Exp Med.

[73] Mandapathil M, Szczepanski MJ, Szajnik M (2009). Increased ectonucleotidase expression and activity in regulatory T cells of patients with head and neck cancer. Clin Cancer Res.

[74] Koyas A, Tucer S, Kayhan M, Savas AC, Akdemir I, Cekic C (2021). Interleukin-7 protects CD8^+^ T cells from adenosine-mediated immunosuppression. Sci Signal.

[75] Cekic C, Linden J (2014). Adenosine A_2A_ receptors intrinsically regulate CD8^+^ T cells in the tumor microenvironment. Cancer Res.

[76] Huang S, Apasov S, Koshiba M, Sitkovsky M (1997). Role of A2a extracellular adenosine receptor-mediated signaling in adenosine-mediated inhibition of T-cell activation and expansion. Blood.

[77] Ohta A, Gorelik E, Prasad SJ (2006). A2A adenosine receptor protects tumors from antitumor T cells. Proc Natl Acad Sci U S A.

[78] Kjaergaard J, Hatfield S, Jones G, Ohta A, Sitkovsky M (2018). A_2A_ adenosine receptor gene deletion or synthetic A_2A_ antagonist liberate tumor-reactive CD8^+^ T cells from tumor-induced immunosuppression. J Immunol.

[79] Newton HS, Chimote AA, Arnold MJ, Wise-Draper TM, Conforti L (2021). Targeted knockdown of the adenosine A_2A_ receptor by lipid NPs rescues the chemotaxis of head and neck cancer memory T cells. Mol Ther Methods Clin Dev.

[80] Ma SR, Deng WW, Liu JF (2017). Blockade of adenosine A2A receptor enhances CD8^+^ T cells response and decreases regulatory T cells in head and neck squamous cell carcinoma. Mol Cancer.

[81] Ohta A, Ohta A, Madasu M (2009). A2A adenosine receptor may allow expansion of T cells lacking effector functions in extracellular adenosine-rich microenvironments^1^. J Immunol.

[82] Moesta AK, Li XY, Smyth MJ (2020). Targeting CD39 in cancer. Nat Rev Immunol.

[83] Sidders B, Zhang P, Goodwin K (2020). Adenosine signaling is prognostic for cancer outcome and has predictive utility for immunotherapeutic response. Clin Cancer Res.

[84] Fong L, Hotson A, Powderly JD (2020). Adenosine 2A receptor blockade as an immunotherapy for treatment-refractory renal cell cancer. Cancer Discov.

[85] Bai Y, Zhang X, Zheng J, Liu Z, Yang Z, Zhang X (2022). Overcoming high level adenosine-mediated immunosuppression by DZD2269, a potent and selective A2aR antagonist. J Exp Clin Cancer Res.

[86] Buisseret L, Rottey S, De Bono JS (2021). Phase 1 trial of the adenosine A_2A_ receptor antagonist inupadenant (EOS-850): update on tolerability, and antitumor activity potentially associated with the expression of the A_2A_ receptor within the tumor. J Clin Oncol.

[87] Lu JC, Zhang PF, Huang XY (2021). Amplification of spatially isolated adenosine pathway by tumor-macrophage interaction induces anti-PD1 resistance in hepatocellular carcinoma. J Hematol Oncol.

[88] Powderly J, Bendell J, Carneiro B (2020). 1073TiP A phase I, first-in-human, multicenter, open-label, dose-escalation study of IPH5201 as monotherapy or in combination with durvalumab ± oleclumab in advanced solid tumours. Ann Oncol.

[89] Pharma I https://clinicaltrials.gov/study/NCT05742607.

[90] Paturel C, Anceriz N, Eyles J (2022). 190P Combination of IPH5201, a blocking antibody targeting the CD39 immunosuppressive pathway, with durvalumab and chemotherapies: preclinical rationale. Immuno-Oncol Technol.

[91] Wainberg Z, Kang YK, Lee KW (2022). Abstract CT015: safety and efficacy of TTX-030, an anti-CD39 antibody, in combination with chemoimmunotherapy for the first line treatment of locally advanced or metastatic gastric/GEJ cancer. Cancer Res.

[92] Bendell J, LoRusso P, Overman M (2023). First-in-human study of oleclumab, a potent, selective anti-CD73 monoclonal antibody, alone or in combination with durvalumab in patients with advanced solid tumors. Cancer Immunol Immunother.

[93] Herbst RS, Majem M, Barlesi F (2022). COAST: an open-label, phase II, Multidrug platform study of durvalumab alone or in combination with oleclumab or monalizumab in patients with unresectable, stage III non-small-cell lung cancer. J Clin Oncol.

[94] Yu W, Sun J, Wang X (2022). Boosting cancer immunotherapy via the convenient A2AR inhibition using a tunable nanocatalyst with light-enhanced activity. Adv Mater.

[95] Wu L, Xie W, Li Y (2022). Biomimetic nanocarriers guide extracellular ATP homeostasis to remodel energy metabolism for activating innate and adaptive immunity system. Adv Sci.

[96] Mao C, Yeh S, Fu J (2022). Delivery of an ectonucleotidase inhibitor with ROS-responsive nanoparticles overcomes adenosine-mediated cancer immunosuppression. Sci Transl Med.

[97] Covarrubias AJ, Perrone R, Grozio A, Verdin E (2021). NAD^+^ metabolism and its roles in cellular processes during ageing. Nat Rev Mol Cell Biol.

[98] Xie N, Zhang L, Gao W (2020). NAD^+^ metabolism: pathophysiologic mechanisms and therapeutic potential. Signal Transduct Target Ther.

[99] Dwivedi S, Rendón-Huerta EP, Ortiz-Navarrete V, Montaño LF (2021). CD38 and regulation of the immune response cells in cancer. J Oncol.

[100] Navas LE, Carnero A (2021). NAD^+^ metabolism, stemness, the immune response, and cancer. Signal Transduct Target Ther.

[101] Yaku K, Okabe K, Hikosaka K, Nakagawa T (2018). NAD metabolism in cancer therapeutics. Front Oncol.

[102] Liu HY, Wang FH, Liang JM (2023). Targeting NAD metabolism regulates extracellular adenosine levels to improve the cytotoxicity of CD8+ effector T cells in the tumor microenvironment of gastric cancer. J Cancer Res Clin Oncol.

[103] Wang Y, Wang F, Wang L (2021). NAD^+^ supplement potentiates tumor-killing function by rescuing defective TUB-mediated NAMPT transcription in tumor-infiltrated T cells. Cell Rep.

[104] Gerner RR, Macheiner S, Reider S (2020). Targeting NAD immunometabolism limits severe graft-versus-host disease and has potent antileukemic activity. Leukemia.

[105] Aswad F, Kawamura H, Dennert G (2005). High sensitivity of CD4^+^CD25^+^ regulatory T cells to extracellular metabolites nicotinamide adenine dinucleotide and ATP: a role for P2X7 receptors. J Immunol.

[106] Hubert S, Rissiek B, Klages K (2010). Extracellular NAD^+^ shapes the Foxp3^+^ regulatory T cell compartment through the ART2-P2X7 pathway. J Exp Med.

[107] Wei Y, Xiang H, Zhang W (2022). Review of various NAMPT inhibitors for the treatment of cancer. Front Pharmacol.

[108] Chini EN (2009). CD38 as a regulator of cellular NAD: a novel potential pharmacological target for metabolic conditions. Curr Pharm Des.

[109] Malavasi F, Deaglio S, Funaro A (2008). Evolution and function of the ADP ribosyl cyclase/CD38 gene family in physiology and pathology. Physiol Rev.

[110] Philip M, Fairchild L, Sun L (2017). Chromatin states define tumour-specific T cell dysfunction and reprogramming. Nature.

[111] Manna A, Kellett T, Aulakh S (2020). Targeting CD38 is lethal to Breg-like chronic lymphocytic leukemia cells and Tregs, but restores CD8^+^ T-cell responses. Blood Adv.

[112] Morandi F, Horenstein AL, Costa F, Giuliani N, Pistoia V, Malavasi F (2018). CD38: a target for immunotherapeutic approaches in multiple myeloma. Front Immunol.

[113] Malavasi F, Deaglio S, Damle R, Cutrona G, Ferrarini M, Chiorazzi N (2011). CD38 and chronic lymphocytic leukemia: a decade later. Blood.

[114] Chen L, Diao L, Yang Y (2018). CD38-mediated immunosuppression as a mechanism of tumor cell escape from PD-1/PD-L1 blockade. Cancer Discov.

[115] Konen JM, Fradette JJ, Gibbons DL (2019). The good, the bad and the unknown of CD38 in the metabolic microenvironment and immune cell functionality of solid tumors. Cells.

[116] Vaisitti T, Audrito V, Serra S (2011). NAD^+^-metabolizing ecto-enzymes shape tumor-host interactions: the chronic lymphocytic leukemia model. FEBS Lett.

[117] Lokhorst HM, Plesner T, Laubach JP (2015). Targeting CD38 with daratumumab monotherapy in multiple myeloma. N Engl J Med.

[118] Moreau P, Dimopoulos MA, Yong K (2020). Isatuximab plus carfilzomib/dexamethasone versus carfilzomib/dexamethasone in patients with relapsed/refractory multiple myeloma: IKEMA Phase III study design. Future Oncol.

[119] Raab MS, Engelhardt M, Blank A (2020). MOR202, a novel anti-CD38 monoclonal antibody, in patients with relapsed or refractory multiple myeloma: a first-in-human, multicentre, phase 1-2a trial. Lancet Haematol.

[120] Ugamraj HS, Dang K, Ouisse LH (2022). TNB-738, a biparatopic antibody, boosts intracellular NAD+ by inhibiting CD38 ecto-enzyme activity. MAbs.

[121] Tarragó MG, Chini CCS, Kanamori KS (2018). A potent and specific CD38 inhibitor ameliorates age-related metabolic dysfunction by reversing tissue NAD^+ ^decline. Cell Metab.

[122] Lagu B, Wu X, Kulkarni S (2022). Orally bioavailable enzymatic inhibitor of CD38, MK-0159, protects against ischemia/reperfusion injury in the murine heart. J Med Chem.

[123] Peyraud F, Guegan JP, Bodet D, Cousin S, Bessede A, Italiano A (2022). Targeting tryptophan catabolism in cancer immunotherapy era: challenges and perspectives. Front Immunol.

[124] Chen CL, Hsu SC, Ann DK, Yen Y, Kung HJ (2021). Arginine signaling and cancer metabolism. Cancers.

[125] Christiansen B, Wellendorph P, Bräuner-Osborne H (2006). Known regulators of nitric oxide synthase and arginase are agonists at the human G-protein-coupled receptor GPRC6A. Br J Pharmacol.

[126] Gilmour SK (2007). Polyamines and nonmelanoma skin cancer. Toxicol Appl Pharmacol.

[127] Cervelli M, Pietropaoli S, Signore F, Amendola R, Mariottini P (2014). Polyamines metabolism and breast cancer: state of the art and perspectives. Breast Cancer Res Treat.

[128] Gerner EW, Bruckheimer E, Cohen A (2018). Cancer pharmacoprevention: targeting polyamine metabolism to manage risk factors for colon cancer. J Biol Chem.

[129] Choudhari SK, Chaudhary M, Bagde S, Gadbail AR, Joshi V (2013). Nitric oxide and cancer: a review. World J Surg Oncol.

[130] Changou CA, Chen YR, Xing L (2014). Arginine starvation-associated atypical cellular death involves mitochondrial dysfunction, nuclear DNA leakage, and chromatin autophagy. Proc Natl Acad Sci U S A.

[131] Cheng CT, Qi Y, Wang YC (2018). Arginine starvation kills tumor cells through aspartate exhaustion and mitochondrial dysfunction. Commun Biol.

[132] Qiu F, Chen YR, Liu X (2014). Arginine starvation impairs mitochondrial respiratory function in ASS1-deficient breast cancer cells. Sci Signal.

[133] Delage B, Luong P, Maharaj L (2012). Promoter methylation of argininosuccinate synthetase-1 sensitises lymphomas to arginine deiminase treatment, autophagy and caspase-dependent apoptosis. Cell Death Dis.

[134] Kim RH, Coates JM, Bowles TL (2009). Arginine deiminase as a novel therapy for prostate cancer induces autophagy and caspase-independent apoptosis. Cancer Res.

[135] Kremer JC, Prudner BC, Lange SES (2017). Arginine deprivation inhibits the warburg effect and upregulates glutamine anaplerosis and serine biosynthesis in ASS1-Deficient cancers. Cell Rep.

[136] Wang W, Zou W (2020). Amino acids and their transporters in T Cell immunity and cancer therapy. Mol Cell.

[137] Crump NT, Hadjinicolaou AV, Xia M (2021). Chromatin accessibility governs the differential response of cancer and T cells to arginine starvation. Cell Rep.

[138] Gannon PO, Godin-Ethier J, Hassler M (2010). Androgen-regulated expression of arginase 1, arginase 2 and interleukin-8 in human prostate cancer. PLoS One.

[139] Tate DJ Jr, Vonderhaar DJ, Caldas YA (2008). Effect of arginase II on L-arginine depletion and cell growth in murine cell lines of renal cell carcinoma. J Hematol Oncol.

[140] Porembska Z, Luboiński G, Chrzanowska A, Mielczarek M, Magnuska J, Barańczyk-Kuźma A (2003). Arginase in patients with breast cancer. Clin Chim Acta.

[141] Chen C, Jiang X, Zhao Z (2023). Inhibition or promotion, the potential role of arginine metabolism in immunotherapy for colorectal cancer. All Life.

[142] Rodriguez PC, Quiceno DG, Zabaleta J (2004). Arginase I production in the tumor microenvironment by mature myeloid cells inhibits T-cell receptor expression and antigen-specific T-cell responses. Cancer Res.

[143] Norian LA, Rodriguez PC, O’Mara LA (2009). Tumor-infiltrating regulatory dendritic cells inhibit CD8^+^ T cell function via _L_-arginine metabolism. Cancer Res.

[144] Ino Y, Yamazaki-Itoh R, Oguro S (2013). Arginase II expressed in cancer-associated fibroblasts indicates tissue hypoxia and predicts poor outcome in patients with pancreatic cancer. PLoS One.

[145] Sippel TR, White J, Nag K (2011). Neutrophil degranulation and immunosuppression in patients with GBM: restoration of cellular immune function by targeting arginase I. Clin Cancer Res.

[146] Ren W, Zhang X, Li W (2020). Circulating and tumor-infiltrating arginase 1-expressing cells in gastric adenocarcinoma patients were mainly immature and monocytic Myeloid-derived suppressor cells. Sci Rep.

[147] Lowe MM, Boothby I, Clancy S (2019). Regulatory T cells use arginase 2 to enhance their metabolic fitness in tissues. JCI Insight.

[148] Gunji Y, Hori S, Aoe T (1994). High frequency of cancer patients with abnormal assembly of the T cell receptor-CD3 complex in peripheral blood T lymphocytes. Jpn J Cancer Res.

[149] Zea AH, Rodriguez PC, Culotta KS (2004). _L_-Arginine modulates CD3ζ expression and T cell function in activated human T lymphocytes. Cell Immunol.

[150] Sosnowska A, Chlebowska-Tuz J, Matryba P (2021). Inhibition of arginase modulates T-cell response in the tumor microenvironment of lung carcinoma. Oncoimmunology.

[151] Rodriguez PC, Quiceno DG, Ochoa AC (2007). L-arginine availability regulates T-lymphocyte cell-cycle progression. Blood.

[152] Geiger R, Rieckmann JC, Wolf T (2016). L-arginine modulates T cell metabolism and enhances survival and anti-tumor activity. Cell.

[153] Czystowska-Kuzmicz M, Sosnowska A, Nowis D (2019). Small extracellular vesicles containing arginase-1 suppress T-cell responses and promote tumor growth in ovarian carcinoma. Nat Commun.

[154] Munder M, Engelhardt M, Knies D (2013). Cytotoxicity of tumor antigen specific human T cells is unimpaired by arginine depletion. PLoS One.

[155] Mussai F, Wheat R, Sarrou E (2019). Targeting the arginine metabolic brake enhances immunotherapy for leukaemia. Int J Cancer.

[156] Aaboe Jørgensen M, Ugel S, Linder Hübbe M (2021). Arginase 1-based immune modulatory vaccines induce anticancer immunity and synergize with anti-PD-1 checkpoint blockade. Cancer Immunol Res.

[157] Satoh Y, Kotani H, Iida Y, Taniura T, Notsu Y, Harada M (2020). Supplementation of l-arginine boosts the therapeutic efficacy of anticancer chemoimmunotherapy. Cancer Sci.

[158] He X, Lin H, Yuan L, Li B (2017). Combination therapy with L-arginine and α-PD-L1 antibody boosts immune response against osteosarcoma in immunocompetent mice. Cancer Biol Ther.

[159] Canale FP, Basso C, Antonini G (2021). Metabolic modulation of tumours with engineered bacteria for immunotherapy. Nature.

[160] Grzybowski MM, Stańczak PS, Pomper P (2022). OATD-02 validates the benefits of pharmacological inhibition of arginase 1 and 2 in cancer. Cancers.

[161] Borek B, Nowicka J, Gzik A (2023). Arginase 1/2 inhibitor OATD-02: From discovery to first-in-man setup in cancer immunotherapy. Mol Cancer Ther.

[162] Pilanc P, Wojnicki K, Roura AJ (2021). A novel oral arginase 1/2 inhibitor enhances the antitumor effect of PD-1 inhibition in murine experimental gliomas by altering the immunosuppressive environment. Front Oncol.

[163] Naing A, Bauer T, Papadopoulos K (2019). Phase I study of the arginase inhibitor INCB001158 (1158) alone and in combination with pembrolizumab (PEM) in patients (Pts) with advanced/metastatic (adv/met) solid tumours. Ann Oncol.

[164] Koyama T, Shimizu T, Matsubara N (2021). MO10-6 Phase 1 study of retifanlimab (anti-PD-1) and INCB001158 (arginase inhibitor), alone or in combination, in solid tumors. Ann Oncol.

[165] Papadopoulos KP, Tsai FYC, Bauer TM (2017). CX-1158-101: a first-in-human phase 1 study of CB-1158, a small molecule inhibitor of arginase, as monotherapy and in combination with an anti-PD-1 checkpoint inhibitor in patients (pts) with solid tumors. J Clin Oncol.

[166] Altman BJ, Stine ZE, Dang CV (2016). From Krebs to clinic: glutamine metabolism to cancer therapy. Nat Rev Cancer.

[167] Wise DR, Thompson CB (2010). Glutamine addiction: a new therapeutic target in cancer. Trends Biochem Sci.

[168] Son J, Lyssiotis CA, Ying H (2013). Glutamine supports pancreatic cancer growth through a KRAS-regulated metabolic pathway. Nature.

[169] Fan J, Kamphorst JJ, Mathew R (2013). Glutamine-driven oxidative phosphorylation is a major ATP source in transformed mammalian cells in both normoxia and hypoxia. Mol Syst Biol.

[170] Durán RV, Oppliger W, Robitaille AM (2012). Glutaminolysis activates Rag-mTORC1 signaling. Mol Cell.

[171] Metallo CM, Gameiro PA, Bell EL (2011). Reductive glutamine metabolism by IDH1 mediates lipogenesis under hypoxia. Nature.

[172] Yoo HC, Park SJ, Nam M (2020). A variant of SLC1A5 is a mitochondrial glutamine transporter for metabolic reprogramming in cancer cells. Cell Metab.

[173] Ishak Gabra MB, Yang Y, Li H (2020). Dietary glutamine supplementation suppresses epigenetically-activated oncogenic pathways to inhibit melanoma tumour growth. Nat Commun.

[174] Nakaya M, Xiao Y, Zhou X (2014). Inflammatory T cell responses rely on amino acid transporter ASCT2 facilitation of glutamine uptake and mTORC1 kinase activation. Immunity.

[175] Sinclair LV, Rolf J, Emslie E, Shi YB, Taylor PM, Cantrell DA (2013). Control of amino-acid transport by antigen receptors coordinates the metabolic reprogramming essential for T cell differentiation. Nat Immunol.

[176] Carr EL, Kelman A, Wu GS (2010). Glutamine uptake and metabolism are coordinately regulated by ERK/MAPK during T lymphocyte activation. J Immunol.

[177] Presnell SR, Spear HK, Durham J, Riddle T, Applegate A, Lutz CT (2020). Correction: differential fuel requirements of human NK cells and human CD8 T cells: glutamine regulates glucose uptake in strongly activated CD8 T cells. Immunohorizons.

[178] Edwards DN, Ngwa VM, Raybuck AL (2021). Selective glutamine metabolism inhibition in tumor cells improves antitumor T lymphocyte activity in triple-negative breast cancer. J Clin Invest.

[179] Wang W, Guo MN, Li N, Pang DQ, Wu JH (2022). Glutamine deprivation impairs function of infiltrating CD8^+^ T cells in hepatocellular carcinoma by inducing mitochondrial damage and apoptosis. World J Gastrointest Oncol.

[180] Nabe S, Yamada T, Suzuki J (2018). Reinforce the antitumor activity of CD8^+^ T cells via glutamine restriction. Cancer Sci.

[181] Fu Q, Xu L, Wang Y (2019). Tumor-associated macrophage-derived interleukin-23 interlinks kidney cancer glutamine addiction with immune evasion. Eur Urol.

[182] Ma G, Liang Y, Chen Y (2020). Glutamine deprivation induces PD-L1 expression via activation of EGFR/ERK/c-Jun signaling in renal cancer. Mol Cancer Res.

[183] Byun JK, Park M, Lee S (2020). Inhibition of glutamine utilization synergizes with immune checkpoint inhibitor to promote antitumor immunity. Mol Cell.

[184] Leone RD, Zhao L, Englert JM (2019). Glutamine blockade induces divergent metabolic programs to overcome tumor immune evasion. Science.

[185] Pons-Tostivint E, Lugat A, Fontenau JF, Denis MG, Bennouna J (2021). STK11/LKB1 modulation of the immune response in lung cancer: from biology to therapeutic impact. Cells.

[186] Aggarwal C, Thompson JC, Chien AL (2020). Baseline plasma tumor mutation burden predicts response to pembrolizumab-based therapy in patients with metastatic non-small cell lung cancer. Clin Cancer Res.

[187] Biton J, Mansuet-Lupo A, Pécuchet N (2018). *TP53*, *STK11*, and *EGFR* mutations predict tumor immune profile and the response to anti-PD-1 in lung adenocarcinoma. Clin Cancer Res.

[188] Skoulidis F, Goldberg ME, Greenawalt DM (2018). *STK11/LKB1 *mutations and PD-1 inhibitor resistance in KRAS-mutant lung adenocarcinoma. Cancer Discov.

[189] Best SA, Gubser PM, Sethumadhavan S (2022). Glutaminase inhibition impairs CD8 T cell activation in *STK11-/Lkb1-*deficient lung cancer. Cell Metab.

[190] Sanderson SM, Gao X, Dai Z, Locasale JW (2019). Methionine metabolism in health and cancer: a nexus of diet and precision medicine. Nat Rev Cancer.

[191] Neidhart M https://www.sciencedirect.com/book/9780124201941/dna-methylation-and-complex-human-disease.

[192] Ouyang Y, Wu Q, Li J, Sun S, Sun S (2020). S-adenosylmethionine: a metabolite critical to the regulation of autophagy. Cell Prolif.

[193] Froese DS, Fowler B, Baumgartner MR (2019). Vitamin B_12_, folate, and the methionine remethylation cycle - biochemistry, pathways, and regulation. J Inherit Metab Dis.

[194] Lückerath K, Lapa C, Albert C (2015). 11C-Methionine-PET: a novel and sensitive tool for monitoring of early response to treatment in multiple myeloma. Oncotarget.

[195] Glaudemans AWJM, Enting RH, Heesters MAAM (2013). Value of ^11^C-methionine PET in imaging brain tumours and metastases. Eur J Nucl Med Mol Imaging.

[196] Wang Z, Yip LY, Lee JHJ (2019). Methionine is a metabolic dependency of tumor-initiating cells. Nat Med.

[197] Zhao L, Su H, Liu X (2022). mTORC1-c-Myc pathway rewires methionine metabolism for HCC progression through suppressing SIRT4 mediated ADP ribosylation of MAT2A. Cell Biosci.

[198] Ulanovskaya OA, Zuhl AM, Cravatt BF (2013). NNMT promotes epigenetic remodeling in cancer by creating a metabolic methylation sink. Nat Chem Biol.

[199] Sinclair LV, Howden AJ, Brenes A (2019). Antigen receptor control of methionine metabolism in T cells. eLife.

[200] Hung MH, Lee JS, Ma C (2021). Tumor methionine metabolism drives T-cell exhaustion in hepatocellular carcinoma. Nat Commun.

[201] Albers E (2009). Metabolic characteristics and importance of the universal methionine salvage pathway recycling methionine from 5'-methylthioadenosine. IUBMB Life.

[202] Li T, Tan YT, Chen YX (2023). Methionine deficiency facilitates antitumour immunity by altering m^6^A methylation of immune checkpoint transcripts. Gut.

[203] Bian Y, Li W, Kremer DM (2020). Cancer SLC43A2 alters T cell methionine metabolism and histone methylation. Nature.

[204] Tripathi P, Kurtulus S, Wojciechowski S (2010). STAT5 is critical to maintain effector CD8^+^ T cell responses. J Immunol.

[205] Xu S, Chaudhary O, Rodríguez-Morales P (2021). Uptake of oxidized lipids by the scavenger receptor CD36 promotes lipid peroxidation and dysfunction in CD8^+^ T cells in tumors. Immunity.

[206] Zhang Y, Kurupati R, Liu L (2017). Enhancing CD8^+^ T cell fatty acid catabolism within a metabolically challenging tumor microenvironment increases the efficacy of melanoma immunotherapy. Cancer Cell.

[207] Ma X, Bi E, Lu Y (2019). Cholesterol Induces CD8^+^ T cell exhaustion in the tumor microenvironment. Cell Metab.

[208] Mollinedo F, Gajate C (2020). Lipid rafts as signaling hubs in cancer cell survival/death and invasion: implications in tumor progression and therapy: thematic review series: biology of lipid rafts. J Lipid Res.

[209] Ridker PM (2014). LDL cholesterol: controversies and future therapeutic directions. Lancet.

[210] Gelissen IC, Harris M, Rye KA (2006). ABCA1 and ABCG1 synergize to mediate cholesterol export to apoA-I. Arterioscler Thromb Vasc Biol.

[211] Cruz ALS, Barreto EA, Fazolini NPB, Viola JPB, Bozza PT (2020). Lipid droplets: platforms with multiple functions in cancer hallmarks. Cell Death Dis.

[212] Yue S, Li J, Lee SY (2014). Cholesteryl ester accumulation induced by PTEN loss and PI3K/AKT activation underlies human prostate cancer aggressiveness. Cell Metab.

[213] Antalis CJ, Arnold T, Rasool T, Lee B, Buhman KK, Siddiqui RA (2010). High ACAT1 expression in estrogen receptor negative basal-like breast cancer cells is associated with LDL-induced proliferation. Breast Cancer Res Treat.

[214] Mayengbam SS, Singh A, Pillai AD, Bhat MK (2021). Influence of cholesterol on cancer progression and therapy. Transl Oncol.

[215] Dong F, Mo Z, Eid W, Courtney KC, Zha X (2014). Akt inhibition promotes ABCA1-mediated cholesterol efflux to ApoA-I through suppressing mTORC1. PLoS One.

[216] Porstmann T, Santos CR, Griffiths B (2008). SREBP activity is regulated by mTORC1 and contributes to Akt-dependent cell growth. Cell Metab.

[217] Li J, Gu D, Lee SS (2016). Abrogating cholesterol esterification suppresses growth and metastasis of pancreatic cancer. Oncogene.

[218] Thysell E, Surowiec I, Hörnberg E (2010). Metabolomic characterization of human prostate cancer bone metastases reveals increased levels of cholesterol. PLoS One.

[219] Lei K, Kurum A, Kaynak M (2021). Cancer-cell stiffening via cholesterol depletion enhances adoptive T-cell immunotherapy. Nat Biomed Eng.

[220] Huang B, Song BL, Xu C (2020). Cholesterol metabolism in cancer: mechanisms and therapeutic opportunities. Nat Metab.

[221] Maxwell KN, Fisher EA, Breslow JL (2005). Overexpression of PCSK9 accelerates the degradation of the LDLR in a post-endoplasmic reticulum compartment. Proc Natl Acad Sci U S A.

[222] Zhang DW, Lagace TA, Garuti R (2007). Binding of proprotein convertase subtilisin/kexin type 9 to epidermal growth factor-like repeat A of low density lipoprotein receptor decreases receptor recycling and increases degradation. J Biol Chem.

[223] Lagace TA, Curtis DE, Garuti R (2006). Secreted PCSK9 decreases the number of LDL receptors in hepatocytes and in livers of parabiotic mice. J Clin Invest.

[224] Poirier S, Mayer G, Poupon V (2009). Dissection of the endogenous cellular pathways of PCSK9-induced low density lipoprotein receptor degradation: evidence for an intracellular route. J Biol Chem.

[225] Gu Y, Lin X, Dong Y (2023). PCSK9 facilitates melanoma pathogenesis via a network regulating tumor immunity. J Exp Clin Cancer Res.

[226] Yuan J, Cai T, Zheng X (2021). Potentiating CD8^+^ T cell antitumor activity by inhibiting PCSK9 to promote LDLR-mediated TCR recycling and signaling. Protein Cell.

[227] Liu X, Bao X, Hu M (2020). Inhibition of PCSK9 potentiates immune checkpoint therapy for cancer. Nature.

[228] Ni W, Mo H, Liu Y (2021). Targeting cholesterol biosynthesis promotes anti-tumor immunity by inhibiting long noncoding RNA SNHG29-mediated YAP activation. Mol Ther.

[229] Lim WJ, Lee M, Oh Y (2021). Statins decrease programmed death-ligand 1 (PD-L1) by Inhibiting AKT and β-Catenin Signaling. Cells.

[230] Choe EJ, Lee CH, Bae JH, Park JM, Park SS, Baek MC (2022). Atorvastatin enhances the efficacy of immune checkpoint therapy and suppresses the cellular and extracellular vesicle PD-L1. Pharmaceutics.

[231] Wang Q, Cao Y, Shen L (2022). Regulation of PD-L1 through direct binding of cholesterol to CRAC motifs. Sci Adv.

[232] Tatsuguchi T, Uruno T, Sugiura Y (2022). Cancer-derived cholesterol sulfate is a key mediator to prevent tumor infiltration by effector T cells. Int Immunol.

[233] Zech T, Ejsing CS, Gaus K (2009). Accumulation of raft lipids in T-cell plasma membrane domains engaged in TCR signalling. EMBO J.

[234] Liu X, Zhao Z, Sun X (2023). Blocking cholesterol metabolism with tumor-penetrable nanovesicles to improve photodynamic cancer immunotherapy. Small Methods.

[235] Lee IK, Song H, Kim H (2020). RORα regulates cholesterol metabolism of CD8^+^ T cells for anticancer immunity. Cancers.

[236] Yang W, Bai Y, Xiong Y (2016). Potentiating the antitumour response of CD8^+^ T cells by modulating cholesterol metabolism. Nature.

[237] You W, Ke J, Chen Y (2022). SQLE, a key enzyme in cholesterol metabolism, correlates with tumor immune infiltration and immunotherapy outcome of pancreatic adenocarcinoma. Front Immunol.

[238] Furuhashi M, Hotamisligil GS (2008). Fatty acid-binding proteins: role in metabolic diseases and potential as drug targets. Nat Rev Drug Discov.

[239] Guillou H, Zadravec D, Martin PG, Jacobsson A (2010). The key roles of elongases and desaturases in mammalian fatty acid metabolism: insights from transgenic mice. Prog Lipid Res.

[240] Carracedo A, Cantley LC, Pandolfi PP (2013). Cancer metabolism: fatty acid oxidation in the limelight. Nat Rev Cancer.

[241] Lou W, Gong C, Ye Z (2022). Lipid metabolic features of T cells in the tumor microenvironment. Lipids Health Dis.

[242] Tomin T, Fritz K, Gindlhuber J (2018). Deletion of adipose triglyceride lipase links triacylglycerol accumulation to a more-aggressive phenotype in A549 lung carcinoma cells. J Proteome Res.

[243] Snaebjornsson MT, Janaki-Raman S, Schulze A (2020). Greasing the wheels of the cancer machine: the role of lipid metabolism in cancer. Cell Metab.

[244] Argilés JM, Busquets S, Stemmler B, López-Soriano FJ (2014). Cancer cachexia: understanding the molecular basis. Nat Rev Cancer.

[245] Nieman KM, Kenny HA, Penicka CV (2011). Adipocytes promote ovarian cancer metastasis and provide energy for rapid tumor growth. Nat Med.

[246] Wang YY, Attané C, Milhas D (2017). Mammary adipocytes stimulate breast cancer invasion through metabolic remodeling of tumor cells. JCI Insight.

[247] Ye H, Adane B, Khan N (2016). Leukemic stem cells evade chemotherapy by metabolic adaptation to an adipose tissue niche. Cell Stem Cell.

[248] Wen YA, Xing X, Harris JW (2017). Adipocytes activate mitochondrial fatty acid oxidation and autophagy to promote tumor growth in colon cancer. Cell Death Dis.

[249] Ringel AE, Drijvers JM, Baker GJ (2020). Obesity shapes metabolism in the tumor microenvironment to suppress anti-tumor immunity. Cell.

[250] Zhang C, Yue C, Herrmann A (2020). STAT3 activation-induced fatty acid oxidation in CD8^+^ T effector cells is critical for obesity-promoted breast tumor growth. Cell Metab.

[251] Patsoukis N, Bardhan K, Chatterjee P (2015). PD-1 alters T-cell metabolic reprogramming by inhibiting glycolysis and promoting lipolysis and fatty acid oxidation. Nat Commun.

[252] Wang Z, Aguilar EG, Luna JI (2019). Paradoxical effects of obesity on T cell function during tumor progression and PD-1 checkpoint blockade. Nat Med.

[253] Manzo T, Prentice BM, Anderson KG (2020). Accumulation of long-chain fatty acids in the tumor microenvironment drives dysfunction in intrapancreatic CD8^+^ T cells. J Exp Med.

[254] Huang SC, Everts B, Ivanova Y (2014). Cell-intrinsic lysosomal lipolysis is essential for alternative activation of macrophages. Nat Immunol.

[255] Wang H, Franco F, Tsui YC (2020). CD36-mediated metabolic adaptation supports regulatory T cell survival and function in tumors. Nat Immunol.

[256] Yan D, Adeshakin AO, Xu M (2019). Lipid metabolic pathways confer the immunosuppressive function of myeloid-derived suppressor cells in tumor. Front Immunol.

[257] Hossain F, Al-Khami AA, Wyczechowska D (2015). Inhibition of fatty acid oxidation modulates immunosuppressive functions of myeloid-derived suppressor cells and enhances cancer therapies. Cancer Immunol Res.

[258] Auciello FR, Bulusu V, Oon C (2019). A stromal lysolipid-autotaxin signaling axis promotes pancreatic tumor progression. Cancer Discov.

[259] Federico L, Jeong KJ, Vellano CP, Mills GB (2016). Autotaxin, a lysophospholipase D with pleomorphic effects in oncogenesis and cancer progression. J Lipid Res.

[260] Matas-Rico E, Frijlink E, van der Haar Àvila I (2021). Autotaxin impedes anti-tumor immunity by suppressing chemotaxis and tumor infiltration of CD8^+^ T cells. Cell Rep.

[261] Mathew D, Kremer KN, Strauch P, Tigyi G, Pelanda R, Torres RM (2019). LPA_5_ is an inhibitory receptor that suppresses CD8 T-cell cytotoxic function via disruption of early TCR signaling. Front Immunol.

[262] Oda SK, Strauch P, Fujiwara Y (2013). Lysophosphatidic acid inhibits CD8 T cell activation and control of tumor progression. Cancer Immunol Res.

[263] Kremer KN, Buser A, Thumkeo D (2022). LPA suppresses T cell function by altering the cytoskeleton and disrupting immune synapse formation. Proc Natl Acad Sci U S A.

[264] Turner JA, Fredrickson MA, D’Antonio M (2023). Lysophosphatidic acid modulates CD8 T cell immunosurveillance and metabolism to impair anti-tumor immunity. Nat Commun.

[265] Deken M, Niewola K, Matas-rico E (2021). 922 A novel autotaxin inhibitor, IOA-289, modulates tumor, immune and stromal cell function and has monotherapy activity in fibrotic cancer models. J Immunother Cancer.

[266] Deken MA, Niewola-Staszkowska K, Peyruchaud O (2023). Characterization and translational development of IOA-289, a novel autotaxin inhibitor for the treatment of solid tumors. Immunooncol Technol.

[267] Xiong Q, Feng D, Wang Z (2022). Fatty acid synthase is the key regulator of fatty acid metabolism and is related to immunotherapy in bladder cancer. Front Immunol.

[268] Wang Q, Tian N, Zhang W (2022). Fatty acid synthase mutations predict favorable immune checkpoint inhibitor outcome and response in melanoma and non-small cell lung cancer patients. Cancers.

[269] Murphy WJ, Longo DL (2019). The surprisingly positive association between obesity and cancer immunotherapy efficacy. JAMA.

[270] Woodall MJ, Neumann S, Campbell K, Pattison ST, Young SL (2020). The effects of obesity on anti-cancer immunity and cancer immunotherapy. Cancers.

[271] McQuade JL, Daniel CR, Hess KR (2018). Association of body-mass index and outcomes in patients with metastatic melanoma treated with targeted therapy, immunotherapy, or chemotherapy: a retrospective, multicohort analysis. Lancet Oncol.

[272] Cortellini A, Bersanelli M, Buti S (2019). A multicenter study of body mass index in cancer patients treated with anti-PD-1/PD-L1 immune checkpoint inhibitors: when overweight becomes favorable. J Immunother Cancer.

[273] Diana A, Wang LM, D'Costa Z (2016). Prognostic value, localization and correlation of PD-1/PD-L1, CD8 and FOXP3 with the desmoplastic stroma in pancreatic ductal adenocarcinoma. Oncotarget.

[274] Shen T, Zhou L, Shen H (2017). Prognostic value of programmed cell death protein 1 expression on CD8+ T lymphocytes in pancreatic cancer. Sci Rep.

[275] Winograd R, Byrne KT, Evans RA (2015). Induction of T-cell immunity overcomes complete resistance to PD-1 and CTLA-4 blockade and improves survival in pancreatic carcinoma. Cancer Immunol Res.

